# Discovery of a diverse cave flora in China

**DOI:** 10.1371/journal.pone.0190801

**Published:** 2018-02-07

**Authors:** Alexandre K. Monro, Nadia Bystriakova, Longfei Fu, Fang Wen, Yigang Wei

**Affiliations:** 1 Herbarium, Royal Botanic Gardens, Kew, London, United Kingdom; 2 IUCN Cave Invertebrate Specialist Group, Gland, Switzerland; 3 Core Research Laboratories, The Natural History Museum, London, United Kingdom; 4 Guangxi Key Laboratory of Plant Conservation and Restoration Ecology in Karst Terrain, Guangxi Institute of Botany, Guangxi Zhuang Autonomous Region and Chinese Academy of Sciences, Guilin, Guangxi, People's Republic of China; University of Waikato, NEW ZEALAND

## Abstract

Few studies document plants in caves. Our field observations of a widespread and seemingly angiosperm-rich cave flora in SW China lead us to test the following hypotheses, 1) SW China caves contain a diverse vascular plant flora, 2) that this is a relic of a largely absent forest type lacking endemic species, and 3) that the light environment plants occupy in caves is not distinct from non-cave habitats. To do so we surveyed 61 caves and used species accumulation curves (SAC) to estimate the total diversity of this flora and used a subsample of 14 caves to characterise the light environment. We used regional floras and existing conservation assessments to evaluate the conservation value of this flora. We used observations on human disturbance within caves to evaluate anthropogenic activities. Four-hundred-and-eighteen vascular plant species were documented with SACs predicting a total diversity of 529–846. Ninety-three percent of the species documented are known karst forest species, 7% are endemic to caves and 81% of the species are angiosperms. We demonstrate that the light environment in caves is distinct to that of terrestrial habitats and that a subset of the flora likely grow in the lowest light levels documented for vascularised plants. Our results suggest that the proportion of species threatened with extinction is like that for the terrestrial habitat and that almost half of the entrance caverns sampled showed signs of human disturbance. We believe that this is the first time that such an extensive sample of cave flora has been undertaken and that such a diverse vascular plant flora has been observed in caves which we predict occurs elsewhere in SE Asia. We argue that the cave flora is an extension of the karst forest understory present prior to catastrophic deforestation in the 20thC. We suggest that within SW China caves serve as both refuges and a valuable source of germplasm for the restoration of karst forest. We also propose that caves represent a distinct habitat for plants that is most similar to that of the forest understory, but distinct with respect to the absence of trees, leaf litter, root mats, higher levels of atmospheric CO2, and lower diurnal and annual variation in temperature and humidity. We highlight tourism, agriculture and the absence of legislated protection of caves as the main current threats to this flora.

## Introduction

Despite scientists’ long fascination with caves [1] their diversity remains poorly documented, studied and understood with respect to both mineral [2] and biological diversity [3]. The earliest documentations of plants in caves were made by Scopoli [4] and Alexander Von Humboldt in the 18thC [5]. Since that time, however, the few studies that have documented the diversity of plants in caves, have focussed on Europe and the Azores [6–10]. Current estimates of global cave species diversity range from fifty to one hundred thousand species [11] but because there is no global estimate for cave numbers and so even establishing broad parameters to the extent and potential diversity of the cave biome is problematic. Regional estimates for the USA, Europe and China combined suggest that there are *ca* 280 thousand terrestrial caves [12–14] which projected globally suggests a figure of *ca* 1.8 million worldwide.

Caves and karst are closely linked. The dissolution of limestone by water being the major mechanism of cave formation which in turn is the major determinant of karst surface features [15]. Globally karst and the associated limestone / dolomite comprises ca 14% of the terrestrial area [16], the most extensive coverage of which is in Southeast Asia and southern China [17]. The limestone karst of SE Asia have been proposed as a biodiversity hotspot [3] and one within which caves represent an important source of species discovery [18].

That many cave dwelling species have low reproductive potential and small population sizes also makes them vulnerable to extinction [19]. Caves are under threat from the destruction of karst [14, 20–22], desertification triggered by catastrophic deforestation [22], disturbance by agriculture [23, 24] and tourism [25]. Caves and the karst within which the majority are found are also of cultural significance and important features of almost 1/3 of World Heritage Properties that have been listed for their natural importance [16]. Despite the above, only in Brazil, Europe and North America is there legislation and management planning designed to conserve caves or biodiversity and their absence from national legislation or conservation planning elsewhere also represents a threat [1, 24, 26].

Current estimates of global cave biodiversity are based on relatively few groups of organisms and little spatial analysis or application of species area curves and have as their main focus, fauna [27]. Even though plants were first documented in caves in the 18thC [5], the fact that several species having been documented as new to science from caves [28–32] and that investigations into adaptations to photosynthesis in subterranean environments have been published [33] there has to date been little documentation of the diversity or extent of cave floras. The presence of a diverse vascular plant flora in cave entrance caverns would strengthen the notion of caves as ecotones [34] and of connecting terrestrial as well as subterranean networks [35]. It would also suggest that angiosperms are able to photosynthesise in lower light environments than previously documented.

**Fig 1:**
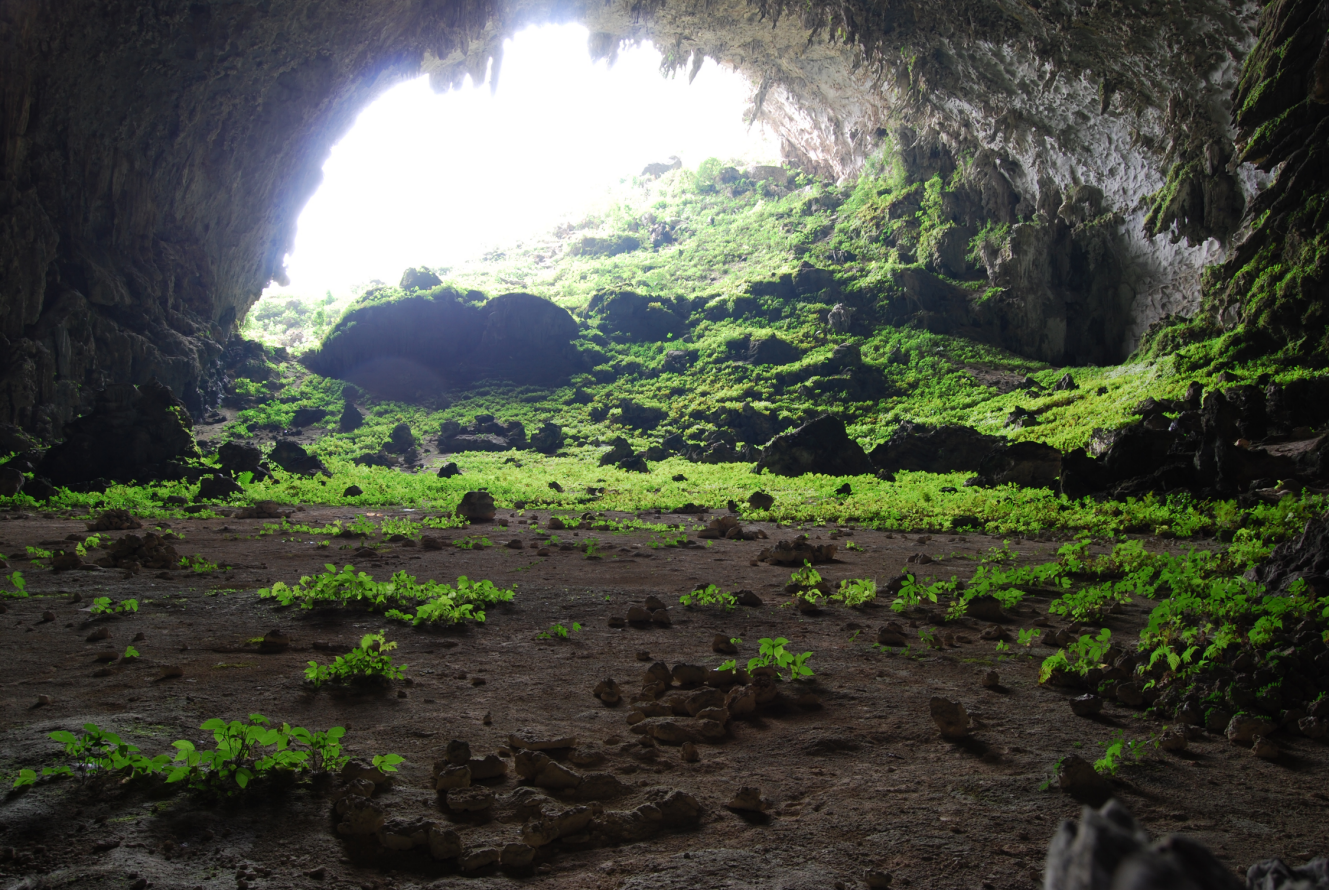
Yangzi cave, Guangxi, China. The cave entrance is approximately 70 m wide, 15 m high and the depth of the entrance cavern supporting vascular plants 170 m. This cave is type locality for 8 vascular plant species.

Fieldwork by the authors suggested a diverse angiosperm flora growing in caves, one of which (Fig 1) is the type locality for eight species of vascular plants. Despite this we could find no documentation of cave floristic diversity for China and few for caves elsewhere, even where broad taxon inventories of caves had been undertaken [5,6]. Where floristic inventories have taken place they have only been of single caves [7–10].

The aim of this research, therefore, was to test the hypotheses that 1) SW China caves contain a diverse angiosperm dominated flora, 2) that this flora is an extension of the neighbouring terrestrial flora and lacks endemic species and, 3) that the light environment occupied by plants in caves is not distinct from terrestrial habitats. In so doing we aimed to document and estimate the diversity of a cave flora for the first time and evaluate its significance to floristic diversity and conservation in the region.

## Materials & methods

### Data collection

#### The study area

The study area comprises 195,921 km2 of early to mid-successional humid subtropical mixed evergreen and deciduous broad-leaved forest [36] growing on a dolomite karst landscape spanning Guangxi, Guizhou and Yunnan in SW China. The landscape and caves are the product of three periods of intensive karstification in the mid Triassic, the Paleogene and the Pleistocene [37]. This karst landscape was subject to virtual total deforestation during China’s ‘Great Leap Forward’ (1958–1961) and ‘Cultural Revolution’ (1966–1976) [38] resulting in much of the vegetation having been converted to a successional scrub, 1/4 to 1/3 of which is currently in the process of desertification [22]. All the caves surveyed were located in successional scrub most often within a broader agricultural landscape.

#### Documenting cave characteristics, surrounding environment and disturbance

**Fig 2:**
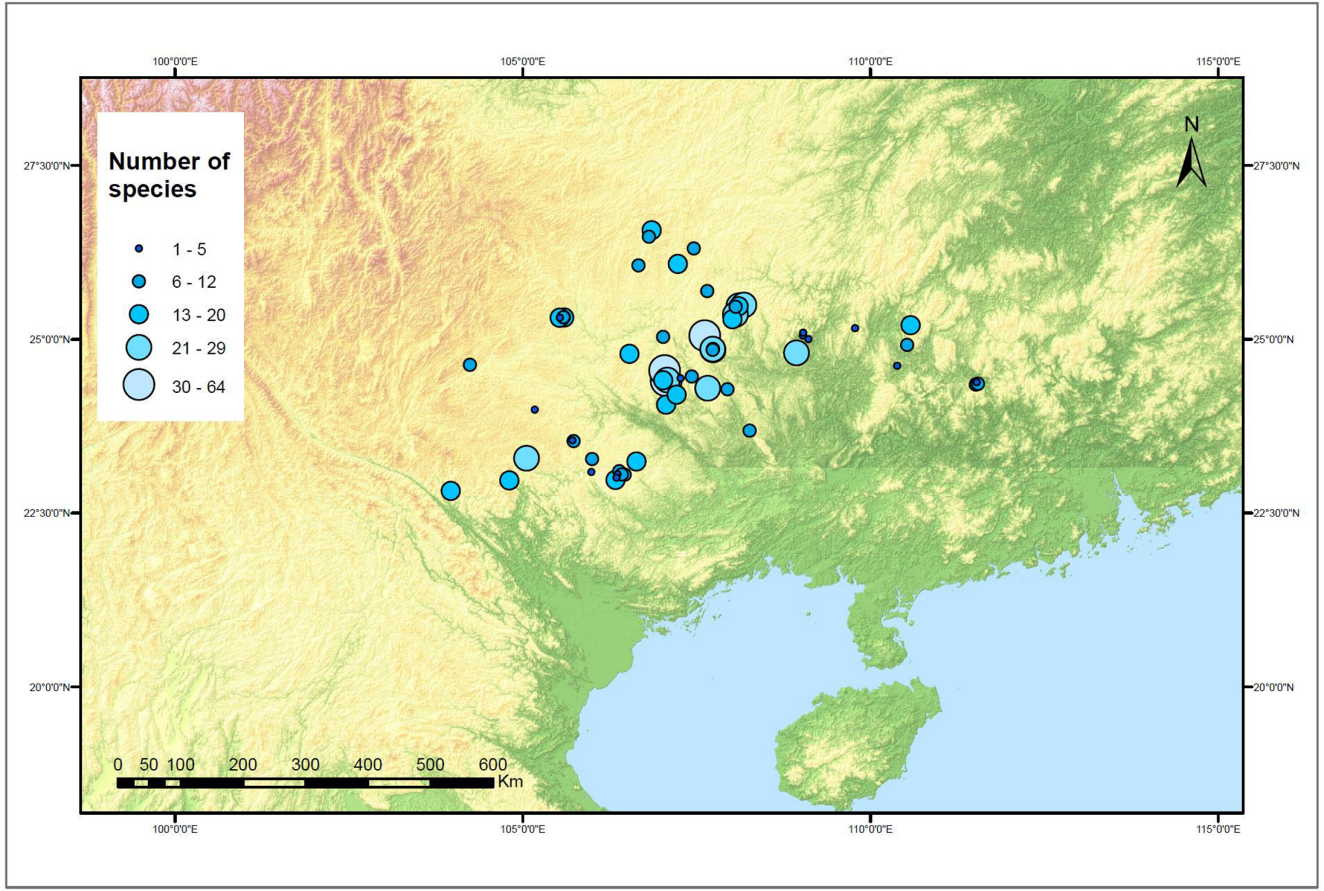
Distribution and species-richness of study caves across SW China. The diameter of the point corresponds to species-richness.

Between 2009 and 2014 we undertook field surveys of 61 caves (Fig 2, S1 Table). The aim of this study was to document the vascular plant diversity which from field observations we knew occurred in caves. We therefore selected caves in a way which we felt would maximise observations of cave vascular plants. We did so by sending a request sent to an informal network of field contacts of the Guangxi Key laboratory of Plant Conservation and Restoration in Karst Terrain. Caves were defined according to Romero [39] comprising an opening that can be entered by humans at least part of which is in total darkness. Caves receive light through their entrances and sink-holes. We used Humphreys’ [40] classification of cave internal space into Entrance, Twilight and Dark zones based on the extent to which light and the external climate impacts on the cave environment. The reason for seeking to assign observations to the entrance or twilight zone was to test whether species-richness and taxonomic composition varied across the light gradient in caves. The cave floor was divided into entrance and based on the asymptotic distribution of light demonstrated by Serena & Meluzzi [9], twilight zones. The entrance zone was defined as the area directly below the opening of a cave and the twilight zone as the area immediately abutting the dark zone to the midpoint between the dark zone and the entrance zone. Applying Humphrey’s definition [40] we defined the dark zone as beginning at the point at which there was no measureable PAR (<0.1 μmol photons m-2 s-1).

At each cave, coordinates, altitude, orientation of the entrance, habitat and disturbance outside and inside of the cave were recorded. To better understand active threats to the entrance caverns we surveyed each entrance cavern for evidence of human disturbance. Disturbance was categorised as: tourism, agriculture- pumping of water, agriculture- storage of livestock, agriculture- cultivation of plants, temporary mausoleums. For a non-random sample of 22 caves we recorded the major dimensions of the entrance and twilight zones of the entrance caverns and produced a sketch-plan of the cave floor.

#### Documenting cave-dwelling vascular plants

Vascular plant populations observed within the footprint of the cave were recorded to species level and assigned to entrance or twilight zone using a PAR meter (see below). This meant that where a cave contained several species of vascular plant, that cave was recorded as having several populations, one of each species. Where identification of plants to species was problematic herbarium collections were made and deposited at IBK, PE and BM.

For each species distributions and habitat information were sourced from the scientific literature [41–44]. Distribution were classified as: 1) within and outside China, 2) endemic to China, 3) endemic to a single province and, 4) known only from a cave. Habitat where the species was encountered outside of caves was classified according to the classification of Hansen et al. [36].

IUCN species conservation assessments were sourced from the China Red List [45, 46] and assessments published with new species descriptions. Because only a small proportion of the species observed growing in caves had been evaluated and that the selection of species for assessment was inconsistent and non-random [47] we decided to evaluate the conservation value of caves using a group of plants, the African Violet family (Gesneriaceae) where threat of extinction had been consistently assessed for all of the species [48].

#### Characterizing the cave light environment

Given that we had observed vascular plants growing in caves we wanted to know whether they were growing in levels of light equivalent to that outside of caves. The aim of this sampling was not to document the PAR levels within each cave but to document the range of light environments that were occupied by plants growing in caves. Given the time that this took and the restricted hours of sampling this was undertaken for a non-random sample of 14 of our study caves. Caves were selected non-randomly using a stratified design that sought to maximise the study area and access rather than individual cave properties such as the number of plants or size of the cave. We characterised the PAR in portions of the cave where plants were observed. PAR was observed between 11:00 and 13:00 hrs. Three point observations of PAR (400–700 nanometer spectrum) were taken parallel to the leaf surface at the entrance and in twilight zones of each sample cave using a calibrated Skye Instruments hand-held Quantum sensor. This resulted in six observation per cave.

### Data analysis

To estimate the total richness of the cave flora and test the hypothesis that cave have diverse flora, we used R version 3.1.2 [49] to generate species accumulation curves (SAC) using, 1) the classic 'random' method [50]; and 2) the ‘exact’ method which uses unconditional standard deviation based on an estimation of the total extrapolated number of species in the survey area [51]. The underlying assumption for each was that each cave had the same floor area, i.e. represented a sampling plot of a standard size. This was not the case meaning that our confidence intervals around the SAC may be larger than calculated. To estimate the extrapolated species richness or the number of unobserved species we used the Chao [52], first and second order Jackknife, and Bootstrap functions.

To test the hypothesis that cave floras are an extension of the surrounding terrestrial flora, we checked whether species observed in the twilight zone of caves were a random subset of the entrance zone flora or had been subject to some degree of filtering as part of the process of twilight zone colonisation. We undertook a crude comparison of the taxonomic composition of the entrance zone and twilight zone flora relative to each ones source flora. The underlying assumption was that species in the entrance zone were drawn from the surrounding regional flora (for a justification see below) and that species in the twilight zone were drawn from the entrance zone flora rather than independently from the regional flora. Our null hypothesis was that the probability of a family or genus colonising the entrance zone should be the same as for it to subsequently colonise the twilight zone. This was tested using a proportion test [53]. We used the taxonomic rank of family as this enabled us to use the Flora of China [41] as a robust baseline, and genus as this enabled us to use regional flora and checklists as robust baselines [42–44] against which we could compare our observed floras.

## Results

### Diversity of cave-dwelling vascular plants

Caves varied in their dimensions. Entrance height’s ranged from 2–120 m, entrance width’s from 4–80 m and depth of the combined entrance and twilight zones from 6–200 m. The average surface area of the cave floor, arbitrarily assuming a rectangular outline was 2253 m^2^. Applied to all 61 of the caves surveyed this gives a total cave floor survey area of 141,916 m^2^. Caves were surveyed at 120 to 1600 m asl.

**Fig 3:**
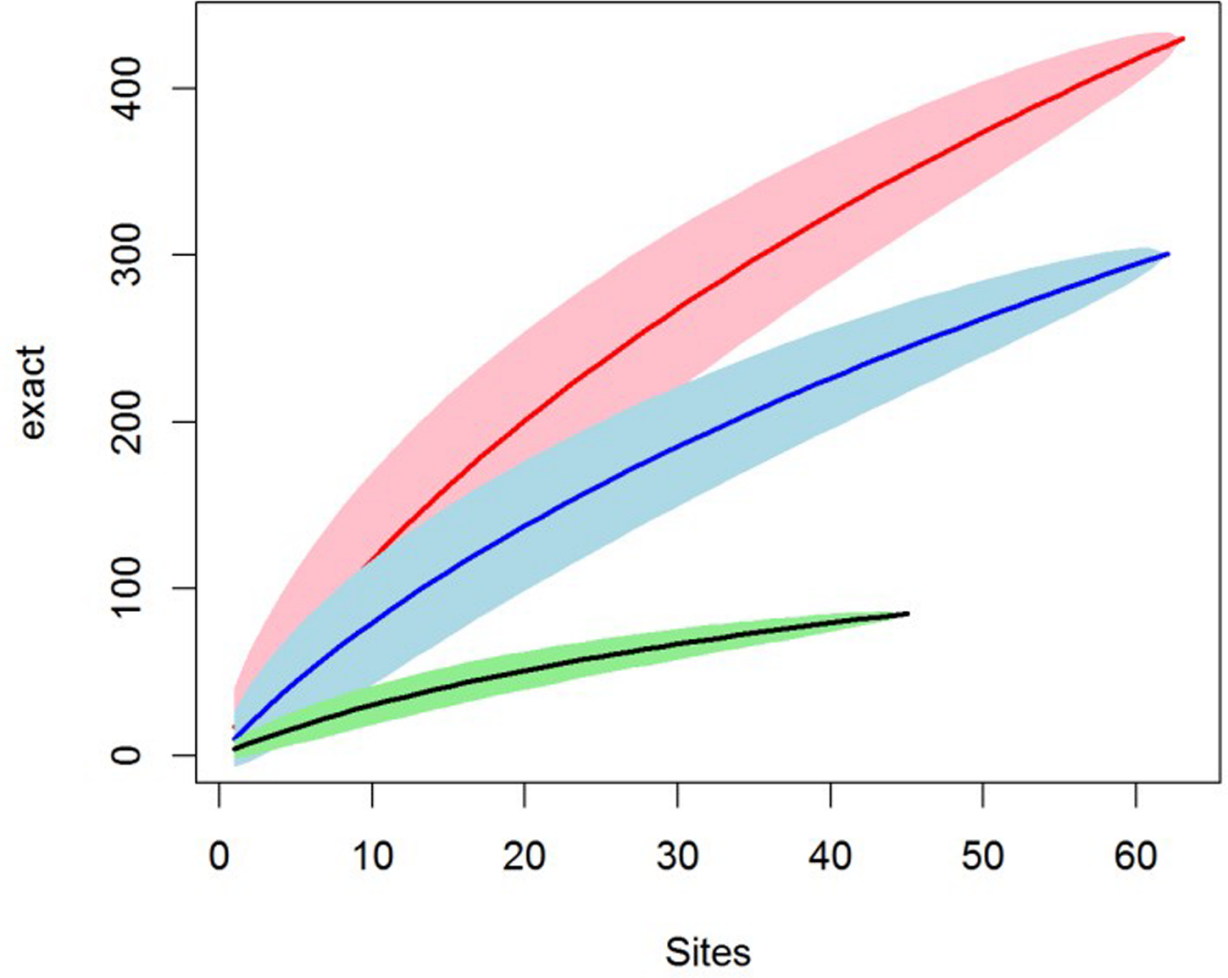
Species accumulation curves. All species (red), angiosperms (blue) and ferns (green).

We recorded 870 populations of 418 species of vascular plants from 186 genera and 83 families (Supporting Information). Species accumulation curves (SAC) and several estimates of species richness (Chao, First order Jackknife, Bootstrap) suggest a total diversity of between 529 and 846 species (Fig 3, S1 Fig). The flora is strongly dominated by understory plant forms and taxa, 88% of the species documented were herbaceous, 8% shrubs or shrublets and 4% vines (lignified) and 80% are angiopserms. Species composition was dominated by the regional karrst forest flora, 93% of the species being also documented from outside of caves within the study area.

The most species-rich families observed were Urticaceae (73 spp), Gesneriaceae (37 spp), Begoniaceae (22 spp), Pteridaceae (20 spp) and Dryopteridaceae (20 spp) and there is strong correlation between these and the most species-rich genera observed, *Elatostema* (42 spp, Urticaceae), *Begonia* (22 spp, Begoniaceae), *Polystichum* (19 spp, Dryopteridaceae), *Primulina* (19 spp, Gesneriaceae) and *Pilea* (13 spp, Urticaceae). Of the 83 families of vascular plants documented in caves, 72 are angiosperms.

The species most frequently observed in caves were *Elatostema cyrtandrifolium* (37% of caves), *Elatostema sublineare* (29% of caves), *Ctenitis rhodolepis* (22%), *Elatostema retrohirtum* (22%) and *Elatostema oblongifolium* (21%). The most frequently surveyed species that have only ever been documented from caves were, *Lysionotus fengshanensis* (9.5%), *Aspidistra cavicola* (9.5%), *Mitreola pingtaoi* (8%), *Lysimachia filipes* (8%) and *Pilea cavernicola* (6%), 254 species were observed only once in caves.

The number of species per cave ranged from 1 to 64 (Fig 2) with an average of 14 species per cave and median of 11. The average number of caves in which each species was observed was 2.06, with a range of 1–23.

### Endemism, conservation value and threats to cave vascular plants

A proportion test undertaken to evaluate whether the assemblage of species observed in the twilight and entrance zones was the product of the same or distinct filtering from the source flora suggests a significant (P = 0.002164) difference. At the rank of plant family there was no significant difference (X-squared = 1.378, df = 1, p-value = 0.2404), whilst at the rank of genus there was a significant difference (X-squared = 6.9275, df = 1, p-value = 0.008488).

The species observed growing in the entrance zone of caves were drawn from ca 1/4 of the plant families present in the region [41] (83 out of a possible 312) and 1/13th of the genera (186 out of a possible 2499). In contrast, the species observed growing in the twilight zone of caves were drawn from 1/8th of the plant families in the region (10 out of a possible 83) and 1/20th of the genera (9 out of a possible 186). These results suggest that there are more barriers to colonising the twilight zone than compared to the entrance zone.

Thirty-one species or 7% of the total cave flora were assessed as endemic to caves having only ever been documented growing in caves (Supporting Information). Seventy-four species (17%) are province endemics and 157 species (37%) are Chinese endemics. Of the 418 species observed growing in the entrance zones of caves, only 15 were observed growing in the twilight zone, none of which were endemic to caves and all of which were species documented from the karst forests of SW China [41].

We sourced existing extinction threat assessments for 48 of the 418 species that we document growing in caves (S2 Table). Of these, 47 (98% of those evaluated) were assessed as vulnerable (VU) to Critically Endangered (CR). We believe that the figure of 98% is likely a biased sample as common widespread species are less likely to be evaluated than rare ones [47]. Using the Gesneriaceae, where all species have been evaluated, as a surrogate we found that of the 43 Gesneriaceae species observed in caves, 56% were assessed as Vulnerable to Critically Endangered, compared to 57% for all habitats in South China [45] (S3 Table).

**Fig 4:**
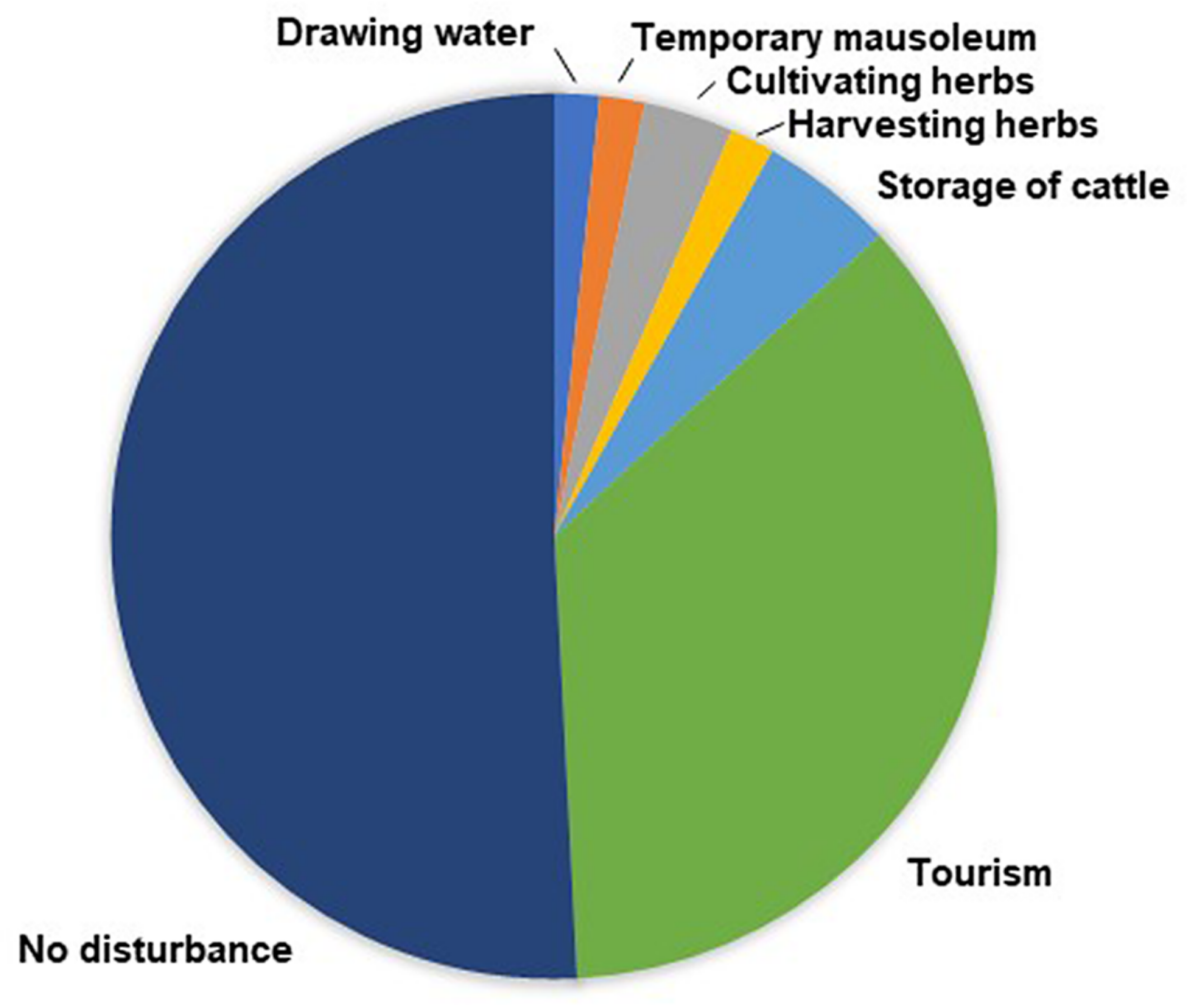
Summary of human disturbance observed in the 61 caves surveyed. Grey (no disturbance), pale blue (tourism), yellow (storage of cattle), pale green (cultivation of herbs), dark grey (harvesting of plants), white (temporary mausoleum) and dark blue (drawing water).

Whilst all caves were observed to be located within a human-modified landscape, we observed that the interior of 30 out of 61 caves (48%) were disturbed by human activity (Fig 4). The most frequent impact was tourism (35% of the caves surveyed), followed by agriculture (10%). This was followed by the use of caves as a water source for nearby communities or as mausoleums (1.5% respectively). The most common agricultural impact observed was the use of caves for the storage of water buffalo at night.

### Light environment in caves

Observations of PAR from a subsample of 14 caves are summarised in Table 1. PAR was significantly higher at the entrance zone (mean = 41.75 μmol photons m-2 s-1) than in the twilight zone (mean = 1.97 μmol photons m-2 s-1). Mean entrance zone PAR for each cave ranged from 3.67–105.57 μmol photons m-2 s-1 and mean twilight zone Par for each cave ranged from 0.35–6.43 μmol photons m-2 s-1.The variances are not equal in the two groups and so the degrees of freedom were not a whole number (the Welch-Satterthwaite correction was applied).

**Table 1:** Summary of PAR observations taken from a subsample of 14 caves. Observation taken parallel to leaf surface at points at which vascular plants were growing. Means are of 3 observations taken in each zone.

Cave ID	Entrance zone mean (μmol photons m-2 s-1)	SD	Twilight zone mean (μmol photons m-2 s-1)	SD
1	68.67	33.69	0.35	0.21
2	57.13	20.50	NA*^a^	NA
3	73.70	23.74	0.55	0.07
5	14.83	2.25	1.43	0.55
8	16.45	1.06	1.77	0.45
10	11.4	8.34	1.50	0.42
11	5.30	0.75	2.23	0.86
21	105.57	12.70	2.83	0.67
31	3.67	0.58	0.47	0.15
32	13.80	6.17	1.23	0.31
34	18.75	3.18	6.43	3.09
61	84.13	17.04	3.43	1.12
62	65.43	2.97	2.70	0.70
63	45.67	24.71	0.70	NA*^b^
**Mean**	**41.75**		**1.97**	
**SD**	**33.74**		**1.66**	

*^a.^ No observations recorded for twilight zone of cave 2

*^b^A single observation recorded for the twilight zone of cave 63

## Discussion

### We document for the first time in Asia a diverse flora associated with caves

We document for the first time in Asia a subterranean angiosperm dominated flora growing in the entrance caverns of cave systems, a proportion of which is restricted to the subterranean habitat (Supporting Information). The large expanse of karst on which this study is focussed extends elsewhere in SE Asia which suggests that such a flora also occurs in Cambodia, Indonesia, Laos, Malaysia, Myanmar, Thailand, Vietnam and Papua New Guinea.

Whilst other studies have documented vascular plants growing in caves in Europe and the Azores [7–10], they document only 30 species of vascular plant, of which 76% are angiosperms. In addition, each study documents the diversity of a single cave. The exception being a survey of multiple caves in the Azores [6] but which documented no vascular plants. The fact that we are the first to document such a widespread and rich flora is surprising given the history of collecting in the region going back three centuries and the numerous angiosperm species described from caves [28–32]. This suggests that either cave biologists in the region have not sought to document plants, or botanists have not sought to collect in caves and we hope that our results will motivate botanists and cave biologists to document vascular plant diversity of caves elsewhere in Asia.

We could not find any other published studies which document the diversity of more than one cave and so support estimates of species turnover and species area curves to be calculated. Of those studies which documented vascular plants in caves, all were for European caves and observed 9 [9], 18 [7], 6 [10], and 5 [8] species respectively. Combined this gives an average diversity of 9.5 species and total diversity of 30 which we feel is significantly different from our observations from 61 caves of an average diversity of 14 species, and total diversity of 418 species.

**Fig 5:**
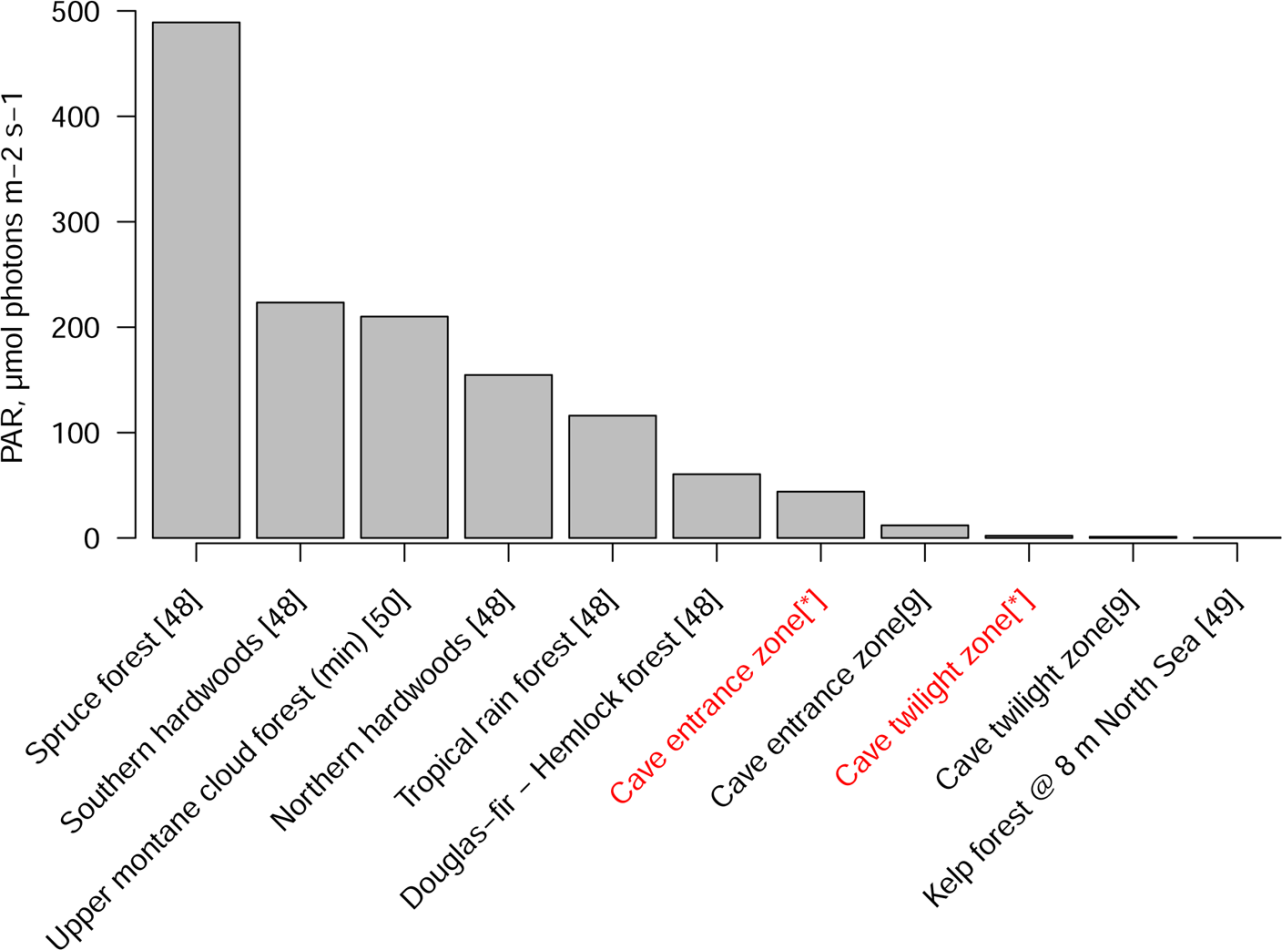
Light levels recorded in the entrance and twilight zones of caves compared to other previously documented low-light vascular plant habitats. Numbers in square brackets refer to reference used to source of data, * refers to this study.

Ferns and spike mosses comprise 20% of the flora that we document and whilst we did not seek to document mosses, surprisingly given the documentation of an exclusively moss flora in Azorean lava tube caves [6], very few were observed growing inside of the caves surveyed. This may be because in the absence of precipitation, whilst pteridophytes have a vascular system which enables them to extract water from the soil and porous rock, bryophytes have only a limited ability to do so [54]. That angiosperms make up such a large proportion of the flora is similar to the results of cave inventories from Europe [7–10] and suggests that angiosperms are better able to colonise low light habitats. It may also reflect the respective terrestrial understory floras which would have abutted cave entrances and we suggest that, would have extended into caves as both habitats being characterised by low levels of PAR (Fig 5). Such co-occurrence of species in caves and the surrounding ecosystem has been widely documented for cave faunas where percentages of species co-occurring range from 82–95% [27, 55]. This hypothesis is supported by the strong dominance (93%) of regional forest understory species in the flora that we document in caves, despite most of this forest having been lost and none being observed adjacent to our study caves. According to this hypothesis, at deforestation species growing in caves persisted, changes in microclimate outside of the cave being buffered by the volume of porous karst within which caves are located. We suggest therefore that the cave flora is a relic of the regional karst forest flora.

Of the flora we document, whilst ferns and spikemosses have previously been associated with low-light environments [7–10, 56], Urticaceae have not. That the Urticaceae genus *Elatostema* were the most species-rich group in both the entrance and twilight zone may reflect their understory habitat and high diversity on karst [57] and so further supports the notion of cave floras as extensions of the terrestrial forest understory.

We document 31 species that are endemic to cave entrance caverns. We believe that it is unlikely that they represent species present but not yet documented in the surrounding regional flora as considerably greater sample effort has been applied to documenting the regional flora [41–44, 48] compared to the cave flora. In addition only five endemic species were observed in the twilight zones of caves and none were restricted to it. Whilst it is conceivable that the cave endemic species represent taxa that have evolved in caves we believe that the most likely explanation for these taxa being restricted to caves is that their populations outside of caves have been lost as a consequence of catastrophic deforestation during the 20thC [38]. The flora’s broad diversity, its dominance by karst understory species, coupled with the observation that cave endemic taxa are associated with the entrance zone rather than the twilight zone strongly suggests that this flora is derived from the regional flora.

### Plant conservation value of caves and threats to cave flora

The karst forests of Guangxi and Guizhou were virtually eliminated during the 1950’s to early 1970’s as part of China’s ‘Great Leap Forward’ and the subsequent ‘Cultural Revolution’ [38, 58]. This has resulted in the conversion of 26–37% of once-forested karst to rocky desert [22] and much of the remainder to a scrub vegetation whose diversity is slightly over ¼ of the original forest [59]. In a deforested landscape, elements of the understory environment such as low light and high humidity would persist only in caves which may function as ‘mini’ refuges for some of the forest understory flora. A function that will likely increase as pressures on the karst landscape increases [3, 60]. Circumstantial support for this scenario is the observation that all 31 cave endemic species were described for the first time after 1981, following both the Great Leap Forward and the Cultural Revolution and so according to this scenario, after their forest habitat had been lost.

If our hypothesis is correct then cave restricted species could provide a means of quantifying the impact of deforestation on understory floras. For example, knowing that the species richness of our study area is *ca* 10,000 [41], of which 418 / 10,000 are able survive and reproduce in caves and observing that 31 species that we believe have lost all their non-cave populations, we could calculate that (31 / 10,000) X (10,000 / 418) or 7.4% of the flora has been lost due to deforestation.

Evaluating the importance of the cave flora for the conservation of threatened species is problematic as the extinction risk of only a small non-random percentage of the plants of Guizhou, Guangxi and Yunnan have been assessed [41, 45]. We found that 11% of the cave flora had been evaluated as Vulnerable to Critically Endangered with extinction which we propose as an absolute minimum estimate for the percentage of the cave flora that is threatened with extinction. Using a complete regional evaluation of the Gesneriaceae we found that the frequency of threatened species in the cave flora is very similar to that for the regional flora (S3 Table). Caves are not therefore likely to comprise a greater proportion of threatened species than the karst forest habitat in which they were located, but they do represent a practical way to maintain populations of understory species that have been impacted where this forest is lost. Not least because access can be relatively easily controlled and conserving cave floras would have little impact on local agriculture or livelihoods. If we are correct that the 31 cave-restricted species we document represent species whose non-cave populations have been lost to deforestation then cave populations also represent a critical source of germplasm, not only for their survival but also for the restoration of karst forest, a current focus for the Chinese Academy of Sciences in Guangxi and Guizhou. Whilst most of the observed cave populations are small, comprising <500 individual, they are however located in caves which are spaced tens or hundreds of km apart and so may represent a broad sample of the specie’s original genetic diversity.

In addition to the absence of legal recognition or legislative protection [3, 18, 24], the mining of karst [3, 60, 61], drought [24] and desertification [22] which have all been identified as major threats to karst and associated cave ecosystems throughout SE Asia, we also document tourism and agriculture impacting the entrance and twilight zones of the caves located in an agricultural landscape (Fig 4). Tourism was the most frequent impact (35% of caves) and consistently associated with compaction and litter. Agriculture resulted in compaction, and the addition of faecal matter, and so represents a profound impact. Similarly, the harvesting of plants as fodder or medicines is likely to have an impact on the cave flora. In contrast, the use of the caves as a water supply and as temporary mausoleums is unlikely to impact the cave flora, although it will impact hydrology and fauna. Mausoleums in particular are situated close to cave entrances. For cultural reasons they preclude tourism and so may well serve to protect many of the larger more charismatic caves.

Given the conservation value of caves, their potential as micro-refugia and sources of germplasm for restoration within SW China’s karst landscape, legislation to protect caves coupled with a Government body to take responsibility for them is urgent at a time of rapid land-use change. Given that the majority of caves and their biota originated in and abutt a once-forested or forested landscape and that the State Forestry Administration’s remit includes the protection and management of populations of wild plants and animals then this would appear to be the most body to protect China’s caves.

### Cave twilight zone, a distinct biome for plants?

Based on their fauna caves have been widely considered distinct biomes [62–64]. Our comparison of entrance and twilight zone assemblages against presumed ‘source’ floras suggests that the twilight zone flora is the product of a distinct filtering which we feel provides evidence for justification for considering the twilight zone a distinct biome for plants under the definition of a biome [65] as a distinct biological community formed in response to a shared physical climate. As with the Tana di Casteltendine cave in Italy [9] we document distinct light levels between the entrance and twilight zone, with the average entrance zone PAR being 20 times that of the twilight zone with both zones exposed to lower PAR than has been recorded on the forest floor [66] (Fig 5). The twilight zone levels of PAR we document are similar to those documented for a single cave by Serena & Meluzzi [9] but below those recorded from terrestrial habitats dominated by vascularized plants [66–69] (Fig 5) where the lowest mean PAR in a review of the literature ranged from 60.5 to 489 μmol photons m-2 s-1 [47] (Fig 5). Combined with our observations of PAR and the documentation of cave climates as highly stable and with low diurnal and seasonal variation [68,70] we suggest that a distinct climate, light regime and the dominance of understory species makes the twilight zones of caves represent a distinct biome for plants. That is a distinct species community lacking endemic species which has formed in response to the shared physical climate of cave entrance caverns. The entrance zones could be considered to be ecotones between karst subtropical moist forest and the twilight zone. Further work to test this hypothesis could include the comparison of species composition within caves at different elevations to that in the neighbouring terrestrial flora.

We have undertaken only the second published multi cave survey of plants and the first undertaken in Asia or the tropics. This documents an angiosperm dominated flora comprising several hundred species, 31 of which are endemic to caves. That such a diverse flora has only now been documented suggests that little attention has been paid to the diversity of entrance caverns, thereby underestimating both the connectivity of caves to the surrounding terrestrial environment and their biodiversity. Both of which could have important implications for the protection and management of what is an increasingly threatened biome [3].

## Conclusions

That the diverse vascular flora we document is strongly dominated by karst forest species leads us to suggest that it is derived from, and a relic of, a now largely absent vegetation lost during the Great Leap Forwards and Cultural Revolution in the 20thC. For this reason, we believe that this flora is of significant value for the conservation of species diversity in SW China and in need of protection from tourism and agriculture. In addition to its value for conservation, this flora suggests that a broad range of vascular plants, especially those from the Urticaceae angiosperm family, can photosynthesise at much lower light levels than hitherto known. We believe that the documentation of this flora establishes a basis for future research on hitherto under studied features of cave biology but also an impetus to document cave flora’s from elsewhere in Asia and the tropics.

## Supporting information

S1 Table Locations and altitudes of study caves. Coordinates are in decimal format and recorded using a Garmin etrex GPS, * denotes used in subsample for characterisation of photosynthetically active radiation in caves.
(DOCX)

S2 Table A preliminary checklist to the cave flora of the study area (SW China). This is based on field inventories of 61 caves in Guizhou, Guangxi and Yunnan, China.
(DOCX)

S3 Table A comparison of the frequency of threatened species in cave and non-cave habitats. Comparison based on the use of a complete assessment of the Gesneriaceae angiosperm family. Source, Gesneriaceae of South China, Nanning: Guangxi Science and Technology Publishing House, Nanning; 2010.
(DOCX)

S1 Fig Species accumulation curves (SAC) with cave floristic dataset (all species). The SAC produced by “exact” method is shown in blue; the SAC produced by “random” method is shown as yellow boxes with outlies as black crosses.
(TIF)
