# Supplementary material for: Discovery of a diverse cave flora in China
Source: PLoS One. 2018 Feb 7;13(2):e0190801. doi: 10.1371/journal.pone.0190801 (PMC5802439; doi:10.1371/journal.pone.0190801)
Supplement: S1 Table — Coordinates are in decimal format and recorded using a Garmin etrex GPS, * denotes used in subsample for characterisation of photosynthetically active radiation in caves. (DOCX) [file pone.0190801.s001.docx]

**S1 Table. Locations and altitudes of study caves.** Coordinates are in decimal format and recorded using a Garmin etrex GPS, * denotes used in subsample for characterisation of photosynthetically active radiation in caves.

| **Cave id** |  | **Province** | **N coordinates** | **E coordinates** | **Altitude** | **Cave orientation** | **Cave entrance height (m)** | **Cave entrance width (m)** | **Cave entrance depth (m)** |
| --- | --- | --- | --- | --- | --- | --- | --- | --- | --- |
| **1** | * | Guangxi | 24°51'17.7 | 107°44'11.4 | 530 | S | 10 | 12 | 42 |
| **2** | * | Guangxi | 24°50'47.3 | 107°44'09.1 | 540 | N | 15 | 6 | 6 |
| **3** | * | Guangxi | 24°50'46.9 | 107°44'14.7 | 550 | N | 10 | 40 | 44 |
| **4** |  | Guangxi | 24°24'26.8 | 107°01'24.6 | 630 | N |  |  |  |
| **5** | * | Guangxi | 24°24'38.5 | 107°04'53.5 | 740 | S | 60 | 80 | 150 |
| **6** |  | Guangxi | 24°24'03.2 | 107°03'51.9 | 550 |  |  |  |  |
| **7** |  | Guizhou | 25°18'23.7 | 105°32'12.8 | 920 | NE |  |  |  |
| **8** | * | Guizhou | 25°18'22.6 | 105°32'03.5 | 980 | NW | 12 | 20 | 25 |
| **9** | * | Guizhou | 25°18'53.8 | 105°35'18.8 | 1040 | NNE | 15 | 35 | 100 |
| **10** | * | Guizhou | 25°18'58.5 | 105°35'46.6 | 900 | W | 30 | 25 | 80 |
| **11** | * | Guizhou | 25°18'45.9 | 105°35'58.8 | 1080 | S | 12 | 25 | 30 |
| **12** |  | Yunnan | 24°37'54.0 | 104°14'43.9 | 1200 | W | 120 | 60 |  |
| **13** |  | Yunnan | 23°59'05.8 | 105°10'37.5 | 1250 | NE |  |  |  |
| **14** |  | Yunnan | 23°32'31.7 | 105°43'00.2 | 770 |  |  |  |  |
| **15** |  | Yunnan | 23°31'55.2 | 105°44'09.2 | 890 | W |  |  |  |
| **16** | * | Guangxi | 23°16'28.1 | 105°59'56.0 | 900 | S | 20 | 20 | 40 |
| **18** |  | Guangxi | 24°12'05.06 | 107°12'54.47 | 263 |  |  |  |  |
| **19** | * | Guangxi | 23°03'21.4 | 106°21'57.6 | 740 | NE | 4 | 2 | 6 |
| **20** | * | Guangxi | 23°02'59.4 | 106°28'12.1 | 740 | N | 7 | 20 | 40 |
| **21** | * | Guangxi | 24°20'49.1 | 111°31'18.2 | 200 | W | 20 | 40 | 60 |
| **22** |  | Guangxi | 24°55'01.04 | 110°31'57.30 | 123 |  |  |  |  |
| **23** |  | Guangxi | 24°36'57.94 | 110°23'20.60 | 156 |  |  |  |  |
| **25** |  | Guangxi | 24°03'24.04 | 107°03'58.05 | 500 |  |  |  |  |
| **26** |  | Guangxi | 25°01'54.9 | 107°01'24.2 | 800 |  |  |  |  |
| **27** |  | Guangxi | 25°05'29.0 | 109°02'13.0 | 150 |  |  |  |  |
| **28** |  | Guangxi | 25°03'04.3 | 109°01'56.3 | 300 |  |  |  |  |
| **29** |  | Guangxi | 25°00'01.3 | 109°06'42.4 | 200 |  |  |  |  |
| **30** |  | Yunnan | 22°57′59 | 104°48′40 | 173 |  |  |  |  |
| **31** | * | Yunnan | 23°17'11.16 | 105°03'22.02 | 1510 | N | 3 | 10 | 40 |
| **32** | * | Yunnan | 22°48'53.94 | 103°58'9.18 | 1600 | NE | 5 | 10 | 35 |
| **33** |  | Guizhou | 26°03'40.21 | 106°40'03.59 | 1242 |  |  |  |  |
| **34** | * | Guizhou | 26°33'51.84 | 106°51'25.92 | 442 |  | 45 | 40 | 200 |
| **35** |  | Guizhou | 25°29'23.04 | 108°11'04.75 | 711 |  |  |  |  |
| **36** |  | Guizhou | 26°18'10.11 | 107°27'44.94 | 947 |  |  |  |  |
| **37** |  | Guizhou | 25°41'33.79 | 107°39'22.76 | 907 |  |  |  |  |
| **38** |  | Guizhou | 25°28'40.92 | 108°06'38.43 | 664 |  |  |  |  |
| **39** |  | Guizhou | 25°17'08.16 | 108°01'10.76 | 680 |  |  |  |  |
| **40** |  | Guizhou | 25°21'09.86 | 108°03'40.44 | 595 |  |  |  |  |
| **41** |  | Guizhou | 26°04'58.80 | 107°14'03.04 | 1055 |  |  |  |  |
| **42** |  | Guizhou | 26°28'24.66 | 106°49'03.26 | 1226 |  |  |  |  |
| **43** |  | Guangxi | 25°02'55.14 | 107°37'13.38 | 209 |  |  |  |  |
| **44** |  | Guangxi | 23°05'57.94 | 106°23'29.33 | 840 |  |  |  |  |
| **45** |  | Guangxi | 22°58'24.72 | 106°20'14.40 | 871 |  |  |  |  |
| **46** |  | Guangxi | 23°03'06.42 | 106°25'35.22 | 706 |  |  |  |  |
| **47** |  | Guangxi | 23°14'16.57 | 106°38'16.56 | 771 |  |  |  |  |
| **48** |  | Guangxi | 24°17'35.49 | 107°39'50.20 | 594 |  |  |  |  |
| **49** |  | Guangxi | 24°32'49.34 | 107°02'31.63 | 484 |  |  |  |  |
| **50** |  | Guangxi | 24°16'43.84 | 107°56'50.26 | 514 |  |  |  |  |
| **51** |  | Guangxi | 25°09'23.88 | 109°47'01.81 | 321 |  |  |  |  |
| **52** |  | Guangxi | 24°27'40.62 | 107°26'04.34 | 561 |  |  |  |  |
| **53** | * | Guangxi | 24°21'33.95 | 111°33'03.07 | 150 | SE | 8 | 8 | 12 |
| **54** |  | Guangxi | 23°41'06.02 | 108°15'59.25 | 306 |  |  |  |  |
| **55** |  | Guangxi | 24°48'05.82 | 108°56'27.82 | 352 |  |  |  |  |
| **56** |  | Guangxi | 25°12'05.58 | 110°34'49.99 | 297 |  |  |  |  |
| **57** |  | Guangxi | 24°26'10.36 | 107°16'21.63 | 640 |  |  |  |  |
| **58** |  | Guangxi | 24°47'22.53 | 106°32'17.51 | 953 |  |  |  |  |
| **59** |  | Guangxi | 23°05'25.28 | 105°59'26.76 | 740 |  |  |  |  |
| **60** | * | Guangxi | 23°00'17.1 | 106°21'04.2 | 740 | NW | 8 | 15 | 25 |
| **61** | * | Guizhou | 25°28'16.62 | 108°06'31.92 | 600 |  | 10 | 30 | 15 |
| **62** | * | Guizhou | 25°27'57.06 | 108°03'56.16 | 660 | NE | 15 | 15 | 25 |
| **63** | * | Guangxi | 24°22'42.5 | 111°31'51.0 | 130 | NNE | 6 | 5 | 10 |
