# Supplementary material for: Discovery of a diverse cave flora in China
Source: PLoS One. 2018 Feb 7;13(2):e0190801. doi: 10.1371/journal.pone.0190801 (PMC5802439; doi:10.1371/journal.pone.0190801)
Supplement: S2 Table — This is based on field inventories of 61 caves in Guizhou, Guangxi and Yunnan, China. (DOCX) [file pone.0190801.s002.docx]

**S2 Table. A preliminary checklist to the cave flora of the study area (SW China).** This is based on field inventories of 63 caves in Guizhou, Guangxi and Yunnan, China.

| **Taxon** | **Family** | **Form** | **Conservation status** | **Distribution** | **Restricted to caves?** | **No. of observations** | **Distribution within caves** |
| --- | --- | --- | --- | --- | --- | --- | --- |
| ***Achyranthes aspera*** L. | Amaranthaceae | herb | not evaluated | Guizhou |  | 4 | entrance zone |
| ***Adiantum capillus-veneris*** L. | Adiantaceae | fern | LC | Guangxi, Guizhou |  | 8 | entrance zone |
| ***Adiantum caudatum*** L. | Adiantaceae | fern | not evaluated | Guangxi |  | 4 | entrance zone |
| ***Adiantum gravesii*** Hance | Adiantaceae | fern | not evaluated | Guangxi |  | 2 | entrance zone |
| ***Adiantum malesianum*** J. Ghatak | Adiantaceae | fern | not evaluated | Guangxi |  | 1 | entrance zone |
| ***Adiantum mariesii*** Baker | Adiantaceae | fern | not evaluated | Guizhou |  | 2 | entrance zone |
| ***Aerva sanguinolenta*** (L.) Blume | Amaranthaceae | herb | not evaluated | Guizhou |  | 1 | entrance zone |
| ***Aeschynanthus austroyunnanensis*** W.T.Wang | Gesneriaceae | herb | not evaluated | Guangxi |  | 1 | entrance zone |
| ***Ajuga macrosperma*** Wall. ex Benth. | Lamiaceae | herb | not evaluated | Guangxi |  | 1 | entrance zone |
| ***Alangium chinense*** (Lour.) Harms | Alangiaceae | shrub | not evaluated | Guangxi |  | 1 | entrance zone |
| ***Alchornea trewioides*** (Benth.) Müll.Arg. | Euphorbiaceae | herb | not evaluated | Guangxi |  | 2 | entrance zone |
| ***Aleuritopteris argentea*** (S.G. Gmel.) Kunze | Sinopteridaceae | fern | not evaluated | Guangxi |  | 1 | entrance zone |
| ***Allantodia griffithii*** (T. Moore) Ching | Athyriaceae | fern | not evaluated | Guizhou |  | 1 | entrance zone |
| ***Allantodia sp1*** | Athyriaceae | fern | not evaluated | Guizhou |  | 1 | entrance zone |
| ***Allocheilos cortusiflorum*** W.T.Wang | Gesneriaceae | herb | EN | Yunnan |  | 1 | entrance zone |
| ***Alocasia macrorrhizos*** (L.) G.Don | Araceae | herb | not evaluated | Guangxi, Guizhou, Yunnan |  | 3 | entrance zone |
| ***Alocasia sp1*** | Araceae | herb | not evaluated | Guangxi |  | 1 | entrance zone |
| ***Amorphophallus konjac*** K.Koch | Araceae | herb | not evaluated | Guangxi |  | 1 | entrance zone |
| ***Amorphophallus sp1*** | Araceae | herb | not evaluated | Guangxi, Guizhou |  | 3 | entrance zone |
| ***Anisopappus chinensis*** (L.) Hook. & Arn. | Asteraceae | herb | not evaluated | Guangxi |  | 1 | entrance zone |
| ***Antenoron filiforme*** (Thunb.) Roberty & Vautier | Polygonaceae | herb | not evaluated | Guizhou |  | 1 | entrance zone |
| ***Aralia chinensis*** L. | Araliaceae | herb | VU | Guangxi |  | 1 | entrance zone |
| ***Ardisia ensifolia*** E.Walker | Primulaceae | herb | not evaluated | Guangxi |  | 1 | entrance zone |
| ***Ardisia maculosa*** Mez | Primulaceae | herb | not evaluated | Guangxi |  | 1 | entrance zone |
| ***Ardisia sp1*** | Primulaceae | shrub | not evaluated | Guangxi |  | 1 | entrance zone |
| ***Arisaema decipiens*** Schott | Araceae | herb | not evaluated | Guangxi |  | 1 | entrance zone |
| ***Arisaema sp1*** | Araceae | herb | not evaluated | Guangxi |  | 1 | entrance zone |
| ***Aristolochia versicolor*** S.M.Hwang | Aristolochiaceae | herb | not evaluated | Guangxi, Yunnan, |  | 2 | entrance zone |
| ***Arthraxon hispidus*** (Thunb.) Makino | Poaceae | herb | not evaluated | Guangxi |  | 2 | entrance zone |
| ***Asarum caudigerum*** Hance | Aristolochiaceae | herb | not evaluated | Guangxi, Guizhou |  | 4 | entrance zone |
| ***Asarum geophilum*** Hemsl. | Aristolochiaceae | herb | not evaluated | Guangxi, Yunnan, |  | 2 | entrance zone |
| ***Asparagus cochinchinensis*** (Lour.) Merr. | Liliaceae | herb | not evaluated | Guizhou |  | 3 | entrance zone |
| ***Aspidistra cavicola*** D. Fang & K.C. Yen | Asparagaceae | herb | CR | Guangxi | only known from caves | 6 | entrance zone |
| ***Aspidistra sp1*** | Asparagaceae | herb | not evaluated | Guangxi |  | 1 | entrance zone |
| ***Aspidistra sp2*** | Asparagaceae | herb | not evaluated | Guangxi |  | 1 | entrance zone |
| ***Aspidistra sp3*** | Asparagaceae | herb | not evaluated | Guizhou |  | 1 | entrance zone |
| ***Aspidistra sp4*** | Asparagaceae | herb | not evaluated | Guangxi, Guizhou |  | 2 | entrance zone |
| ***Asplenium coenobiale*** Hance | Aspleniaceae | fern | not evaluated | Guizhou |  | 1 | entrance & twilight zone |
| ***Asplenium cornutissimum*** X.C.Zhang & R.H.Jiang | Aspleniaceae | fern | not evaluated | Guangxi | only known from caves | 1 | entrance zone |
| ***Asplenium fuscipes*** Baker | Aspleniaceae | fern | not evaluated | Guangxi, Guizhou |  | 2 | entrance zone |
| ***Asplenium sp1*** | Aspleniaceae | fern | not evaluated | Guangxi |  | 3 | entrance zone |
| ***Asplenium sp2*** | Aspleniaceae | fern | not evaluated | Guangxi |  | 1 | entrance zone |
| ***Asplenium sp3*** | Aspleniaceae | fern | not evaluated | Guangxi |  | 1 | entrance zone |
| ***Asplenium sp4*** | Aspleniaceae | fern | not evaluated | Guangxi, Guizhou |  | 2 | entrance zone |
| ***Asplenium sp5*** | Aspleniaceae | fern | not evaluated | Guangxi |  | 1 | entrance zone |
| ***Asplenium unilaterale*** Lam. | Aspleniaceae | fern | not evaluated | Guizhou |  | 3 | entrance zone |
| ***Bauhinia aurea*** H.Lév. | Fabaceae | Liana | not evaluated | Guangxi |  | 1 | entrance zone |
| ***Begonia arachnoidea*** C.I Peng, Yan Liu & S.M.Ku | Begoniaceae | herb | not evaluated | Guangxi |  | 1 | entrance zone |
| ***Begonia asteropyrifolia*** Y.M.Shui & W.H.Chen | Begoniaceae | herb | not evaluated | Guangxi |  | 2 | entrance zone |
| ***Begonia balansana*** Gagnep. | Begoniaceae | herb | not evaluated | Guangxi |  | 2 | entrance zone |
| ***Begonia bamaensis*** Yan Liu & C.I Peng | Begoniaceae | herb | not evaluated | Guangxi | only known from caves | 1 | entrance zone |
| ***Begonia biflora*** T.C.Ku | Begoniaceae | herb | not evaluated | Yunnan |  | 2 | entrance zone |
| ***Begonia cavaleriei*** H.Lév. | Begoniaceae | herb | VU | Guangxi, Guizhou, Yunnan |  | 12 | entrance zone |
| ***Begonia chingii*** Irmsch. | Begoniaceae | herb | not evaluated | Guangxi, Guizhou |  | 6 | entrance zone |
| ***Begonia curvicarpa*** S.M.Ku, C.I Peng & Yan Liu | Begoniaceae | herb | not evaluated | Guangxi |  | 1 | entrance zone |
| ***Begonia debaoensis*** C.I Peng, Yan Liu & S.M.Ku | Begoniaceae | herb | not evaluated | Guangxi | only known from caves | 1 | entrance zone |
| ***Begonia edulis*** H.Lév. | Begoniaceae | herb | not evaluated | Guangxi, Guizhou |  | 6 | entrance & twilight zone |
| ***Begonia fimbristipula*** Hance | Begoniaceae | herb | not evaluated | Guangxi |  | 1 | entrance zone |
| ***Begonia grandis*** Dryand. | Begoniaceae | herb | not evaluated | Guangxi |  | 1 | entrance zone |
| ***Begonia leprosa*** Hance | Begoniaceae | herb | not evaluated | Guangxi |  | 1 | entrance zone |
| ***Begonia luochengensis*** S.M.Ku, C.I Peng & Yan Liu | Begoniaceae | herb | not evaluated | Guangxi |  | 1 | entrance zone |
| ***Begonia palmata*** D. Don | Begoniaceae | herb | not evaluated | Guizhou |  | 1 | entrance zone |
| ***Begonia parvula*** H. Lév. & Vaniot | Begoniaceae | herb | not evaluated | Guizhou |  | 1 | entrance zone |
| ***Begonia picturata*** Yan Liu, S.M.Ku & C.I Peng | Begoniaceae | herb | not evaluated | Guangxi |  | 2 | entrance zone |
| ***Begonia retinervia*** D. Fang, D.H.Qin & C.I. Peng | Begoniaceae | herb | not evaluated | Guangxi |  | 1 | entrance zone |
| ***Begonia smithiana*** T.T.Yu ex Irmsch. | Begoniaceae | herb | not evaluated | Guangxi |  | 1 | entrance zone |
| ***Begonia sp1*** | Begoniaceae | herb | not evaluated | Yunnan |  | 1 | entrance zone |
| ***Begonia sp2*** | Begoniaceae | herb | not evaluated | Guizhou |  | 1 | entrance zone |
| ***Begonia wangii*** T.T.Yu | Begoniaceae | herb | not evaluated | Yunnan |  | 1 | entrance zone |
| ***Berchemia polyphylla*** Wall. ex M.A.Lawsen | Rhamnaceae | shrub | not evaluated | Guangxi |  | 2 | entrance zone |
| ***Bischofia polycarpa*** (H.Lév.) Airy Shaw | Euphorbiaceae | shrub | not evaluated | Guangxi |  | 2 | entrance zone |
| ***Blechnum sp1*** | Blechnaceae | fern | not evaluated | Guangxi, Guizhou |  | 2 | entrance zone |
| ***Blumea balsamifera*** (L.) DC. | Asteraceae | herb | not evaluated | Guangxi |  | 1 | entrance zone |
| ***Blumea lacera*** (Burm.f.) DC. | Asteraceae | herb | not evaluated | Guangxi |  | 3 | entrance zone |
| ***Blumea martiniana*** Vaniot | Asteraceae | herb | not evaluated | Guangxi |  | 1 | entrance zone |
| ***Blumea megacephala*** (Randeria) C.T.Chang & C.H.Yu ex Y.Ling | Asteraceae | herb | not evaluated | Guangxi, Guizhou |  | 3 | entrance zone |
| ***Boehmeria dolichostachya*** W.T.Wang | Urticaceae | herb | not evaluated | Guangxi, Guizhou |  | 3 | entrance zone |
| ***Boehmeria penduliflora*** Wedd. ex D.G.Long | Urticaceae | herb | not evaluated | Guangxi |  | 3 | entrance zone |
| ***Boehmeria silvestrii*** (Pamp.) W.T. Wang | Urticaceae | shrub | not evaluated | Guangxi, Guizhou |  | 6 | entrance zone |
| ***Boehmeria sp1*** | Urticaceae | herb | not evaluated | Guangxi |  | 1 | entrance zone |
| ***Bonia saxatilis*** (L.C.Chia, H.L.Fung & Y.L.Yang) N.H.Xia | Poaceae | herb | not evaluated | Guangxi |  | 1 | entrance zone |
| ***Brandisia sp1*** | Scrophulariaceae | Liana | not evaluated | Guangxi |  | 1 | entrance zone |
| ***Broussonetia kaempferi*** Siebold | Moraceae | Liana | not evaluated | Guangxi |  | 1 | entrance zone |
| ***Broussonetia kazinoki*** Siebold | Moraceae | shrub | not evaluated | Guangxi |  | 1 | entrance zone |
| ***Buddleja lindleyana*** Fortune | Buddlejaceae | herb | not evaluated | Guangxi |  | 1 | entrance zone |
| ***Calamus simplicifolius*** C.F. Wei | Arecaceae | Liana | not evaluated | Guangxi |  | 1 | entrance zone |
| ***Calcareoboea coccinea*** C.Y.Wu | Gesneriaceae | herb | not evaluated | Guangxi |  | 1 | entrance zone |
| ***Canscora lucidissima*** (H.Lév. & Vaniot) Thiv | Gentianaceae | herb | not evaluated | Guangxi |  | 1 | entrance zone |
| ***Carex cruciata*** Wahlenb. | Cyperaceae | herb | not evaluated | Guangxi |  | 1 | entrance zone |
| ***Carex prainii*** Kük. | Cyperaceae | herb | not evaluated | Guizhou |  | 1 | entrance zone |
| ***Caryota maxima*** Blume ex Mart. | Arecaceae | shrub | not evaluated | Guangxi |  | 1 | entrance zone |
| ***Celtis sinensis*** Pers. | Ulmaceae | shrub | not evaluated | Yunnan |  | 1 | entrance zone |
| ***Chrysosplenium sp1*** | Saxifragaceae | herb | not evaluated | Guangxi, Guizhou |  | 2 | entrance zone |
| ***Cissampelopsis spelaeicola*** (Vaniot) C.Jeffrey & Y.L.Chen | Asteraceae | herb | not evaluated | Guangxi, Guizhou |  | 2 | entrance zone |
| ***Clausena anisata*** (Willd.) Hook.f. ex Benth. | Rutaceae | herb | not evaluated | Guangxi |  | 1 | entrance zone |
| ***Clematis sp1*** | Ranunculaceae | herb | not evaluated | Guangxi |  | 1 | entrance zone |
| ***Cochlearia henryi*** (Oliv.) O.E. Schulz | Brassicaceae | herb | not evaluated | Guangxi, Yunnan, |  | 2 | entrance zone |
| ***Colysis elliptica*** (Thunb.) Ching | Polypodiaceae | fern | not evaluated | Yunnan |  | 1 | entrance zone |
| ***Colysis sp1*** | Polypodiaceae | fern | not evaluated | Guizhou |  | 1 | entrance zone |
| ***Coniogramme procera*** Fée | Hemionitidaceae | fern | not evaluated | Guizhou |  | 1 | entrance zone |
| ***Corydalis saxicola*** Bunting | Papaveraceae | herb | not evaluated | Guangxi |  | 3 | entrance zone |
| ***Croton euryphyllus*** W.W.Sm. | Euphorbiaceae | herb | not evaluated | Guangxi |  | 2 | entrance zone |
| ***Cryptotaenia sp1*** | Apiaceae | herb | not evaluated | Guangxi |  | 1 | entrance zone |
| ***Ctenitis rhodolepis*** (C.B. Clarke) Ching | Aspidiaceae | fern | not evaluated | Guangxi, Guizhou, Yunnan |  | 14 | entrance zone |
| ***Ctenitopsis subsageniaca*** Ching | Aspidiaceae | fern | not evaluated | Guangxi, Guizhou |  | 3 | entrance zone |
| ***Cyclosorus aridus*** (D. Don) Tagawa | Thelypteridaceae | fern | not evaluated | Guangxi, Guizhou |  | 2 | entrance zone |
| ***Cyclosorus interruptus*** (Willd.) H. Itô | Thelypteridaceae | fern | LC | Guizhou |  | 1 | entrance zone |
| ***Cyrtogonellum caducum*** Ching | Dryopteridaceae | fern | not evaluated | Guizhou |  | 1 | entrance zone |
| ***Cyrtogonellum sp1*** | Dryopteridaceae | fern | not evaluated | Guizhou |  | 1 | entrance zone |
| ***Cyrtomium caryotideum*** (Wall. ex Hook. & Grev.) C. Presl | Dryopteridaceae | fern | not evaluated | Guangxi, Guizhou |  | 2 | entrance zone |
| ***Cyrtomium fortunei*** J. Sm. | Dryopteridaceae | fern | not evaluated | Guizhou |  | 1 | entrance zone |
| ***Cyrtomium sp1*** | Dryopteridaceae | fern | not evaluated | Guangxi |  | 2 | entrance zone |
| ***Decaneuropsis chingiana*** (Hand.-Mazz.) H.Rob. & Skvarla | Asteraceae | herb | not evaluated | Guangxi |  | 1 | entrance zone |
| ***Deparia boryana*** (Willd.) M. Kato | Athyriaceae | fern | not evaluated | Guizhou |  | 1 | entrance zone |
| ***Deparia okuboana*** (Makino) M. Kato | Athyriaceae | fern | not evaluated | Guizhou |  | 1 | entrance zone |
| ***Didymocarpus niveolanosus*** D. Fang & W.T. Wang | Gesneriaceae | herb | not evaluated | Guangxi |  | 1 | entrance zone |
| ***Diospyros saxatilis*** S.K.Lee | Ebenaceae | shrub | not evaluated | Guangxi |  | 1 | entrance zone |
| ***Diploclisia glaucescens*** (Blume) Diels | Menispermaceae | herb | not evaluated | Guangxi |  | 1 | entrance zone |
| ***Disporopsis fuscopicta*** Hance | Asparagaceae | herb | not evaluated | Guizhou |  | 1 | entrance zone |
| ***Dolichopetalum kwangsiensis*** Tsiang | Asclepiadaceae | Liana | not evaluated | Guangxi |  | 1 | entrance zone |
| ***Drymaria cordata*** (L.) Willd. ex Schult. | Caryophyllaceae | herb | not evaluated | Guangxi |  | 1 | entrance zone |
| ***Dryopteris atrata*** (Wall. ex Kunze) Ching | Dryopteridaceae | fern | not evaluated | Guizhou |  | 1 | entrance zone |
| ***Echinacanthus lofuensis*** (H.Lév.) J.R.I.Wood | Acanthaceae | herb | not evaluated | Guangxi |  | 1 | entrance zone |
| ***Elatostema acuminatum*** (Poir.) Brongn. | Urticaceae | herb | not evaluated | Guangxi |  | 1 | entrance zone |
| ***Elatostema asterocephalum*** W.T.Wang | Urticaceae | herb | not evaluated | Guangxi |  | 4 | entrance zone |
| ***Elatostema atroviride*** W.T.Wang | Urticaceae | herb | not evaluated | Guangxi, Guizhou, Yunnan |  | 3 | entrance zone |
| ***Elatostema backeri*** H.Schroet. | Urticaceae | herb | not evaluated | Yunnan |  | 1 | entrance & twilight zone |
| ***Elatostema balansae*** Gagnep. | Urticaceae | herb | not evaluated | Guangxi, Guizhou, Yunnan |  | 11 | entrance zone |
| ***Elatostema bamaense*** W.T.Wang & Y.G.Wei | Urticaceae | herb | not evaluated | Guangxi | only known from caves | 1 | entrance zone |
| ***Elatostema binatum*** W.T.Wang & Y.G.Wei | Urticaceae | herb | not evaluated | Guangxi | only known from caves | 3 | entrance zone |
| ***Elatostema boehmerioides*** W.T.Wang | Urticaceae | herb | not evaluated | Yunnan |  | 1 | entrance zone |
| ***Elatostema celingense*** W.T.Wang, Y.G.Wei & A.K.Monro | Urticaceae | herb | not evaluated | Guangxi | only known from caves | 3 | entrance zone |
| ***Elatostema coriaceifolium*** W.T.Wang | Urticaceae | herb | not evaluated | Guangxi, Guizhou |  | 1 | entrance zone |
| ***Elatostema cyrtandrifolium*** (Zoll. & Moritzi) Miq. | Urticaceae | herb | not evaluated | Guangxi, Guizhou, Yunnan |  | 23 | entrance & twilight zone |
| ***Elatostema densistriolatum*** W.T.Wang & Z.Y.Wu | Urticaceae | herb | not evaluated | Yunnan | only known from caves | 1 | entrance zone |
| ***Elatostema fengshanense*** W.T.Wang & Y.G.Wei | Urticaceae | herb | not evaluated | Guangxi |  | 5 | entrance zone |
| ***Elatostema gyrocephalum*** W.T.Wang & Y.G.Wei | Urticaceae | herb | not evaluated | Guangxi | only known from caves | 1 | entrance zone |
| ***Elatostema hekouense*** W.T.Wang | Urticaceae | herb | not evaluated | Yunnan |  | 1 | entrance zone |
| ***Elatostema hezhouense*** W.T.Wang, Y.G.Wei & A.K.Monro | Urticaceae | herb | not evaluated | Guangxi | only known from caves | 1 | entrance zone |
| ***Elatostema ichangense*** H.Schroet. | Urticaceae | herb | not evaluated | Guizhou |  | 1 | entrance zone |
| ***Elatostema involucratum*** Franch. & Sav. | Urticaceae | herb | not evaluated | Guizhou |  | 2 | entrance zone |
| ***Elatostema jingxiense*** W.T. Wang & Y.G. Wei | Urticaceae | herb | not evaluated | Guangxi | only known from caves | 1 | entrance zone |
| ***Elatostema lineolatum var. majus*** Wight | Urticaceae | herb | not evaluated | Yunnan |  | 1 | entrance zone |
| ***Elatostema longibracteatum*** W.T.Wang | Urticaceae | herb | not evaluated | Yunnan |  | 1 | entrance zone |
| ***Elatostema longistipulum*** Hand.-Mazz. | Urticaceae | herb | not evaluated | Guangxi, Guizhou |  | 4 | entrance zone |
| ***Elatostema lungzhouense*** W.T.Wang | Urticaceae | herb | not evaluated | Guangxi |  | 1 | entrance zone |
| ***Elatostema macintyrei*** Dunn | Urticaceae | herb | not evaluated | Guangxi,  Yunnan |  | 4 | entrance zone |
| ***Elatostema megacephalum*** W.T.Wang | Urticaceae | herb | not evaluated | Yunnan |  | 1 | entrance zone |
| ***Elatostema melanophyllum*** W.T.Wang | Urticaceae | herb | not evaluated | Yunnan |  | 1 | entrance zone |
| ***Elatostema monandrum*** (Buch.-Ham. ex D.Don) H.Hara | Urticaceae | herb | not evaluated | Guizhou |  | 1 | entrance zone |
| ***Elatostema multicaule*** W.T.Wang, Y.G.Wei & A.K.Monro | Urticaceae | herb | not evaluated | Yunnan |  | 1 | entrance zone |
| ***Elatostema myrtillus*** (H.Lév.) Hand.-Mazz. | Urticaceae | herb | not evaluated | Guangxi, Guizhou |  | 3 | entrance & twilight zone |
| ***Elatostema nanchuanense*** W.T.Wang | Urticaceae | herb | not evaluated | Guangxi, Guizhou, Yunnan |  | 1 | entrance zone |
| ***Elatostema oblongifolium*** S.H.Fu | Urticaceae | herb | not evaluated | Guangxi, Guizhou, Yunnan |  | 13 | entrance zone |
| ***Elatostema obscurinerve*** W.T.Wang | Urticaceae | herb | not evaluated | Guangxi, Guizhou |  | 1 | entrance & twilight zone |
| ***Elatostema pycnodontum*** W.T.Wang | Urticaceae | herb | not evaluated | Guangxi, Guizhou, Yunnan |  | 4 | entrance zone |
| ***Elatostema quinquecostatum*** W.T.Wang | Urticaceae | herb | not evaluated | Yunnan |  | 1 | entrance zone |
| ***Elatostema ramosum*** W.T.Wang | Urticaceae | herb | not evaluated | Guangxi |  | 1 | entrance zone |
| ***Elatostema retrohirtum*** Dunn | Urticaceae | herb | not evaluated | Guangxi, Guizhou, Yunnan |  | 14 | entrance zone |
| ***Elatostema scaposum*** Q.Lin & L.D.Duan | Urticaceae | herb | not evaluated | Guizhou | only known from caves | 2 | entrance zone |
| ***Elatostema sp1*** | Urticaceae | herb | not evaluated | Guangxi, Guizhou |  | 2 | entrance zone |
| ***Elatostema sp2*** | Urticaceae | herb | not evaluated | Guizhou |  | 1 | entrance zone |
| ***Elatostema sublineare*** W.T.Wang | Urticaceae | herb | not evaluated | Guangxi, Guizhou |  | 18 | entrance & twilight zone |
| ***Elatostema tenuinerve*** W.T.Wang & Y.G.Wei | Urticaceae | herb | not evaluated | Guangxi | only known from caves | 1 | entrance zone |
| ***Elatostema xichouense*** W.T.Wang | Urticaceae | herb | not evaluated | Guizhou |  | 1 | entrance zone |
| ***Epithema carnosum*** Benth. | Gesneriaceae | herb | not evaluated | Guangxi, Guizhou, Yunnan |  | 6 | entrance zone |
| ***Equisetum ramosissimum*** Desf. | Equisetaceae | herb | not evaluated | Guangxi |  | 1 | entrance zone |
| ***Eriophorum comosum*** (Wall.) Nees | Cyperaceae | herb | not evaluated | Guangxi |  | 2 | entrance zone |
| ***Euonymus fortunei*** (Turcz.) Hand.-Mazz. | Celastraceae | Liana | not evaluated | Guangxi |  | 1 | entrance zone |
| ***Fatoua villosa*** (Thunb.) Nakai | Moraceae | herb | not evaluated | Guangxi |  | 1 | entrance zone |
| ***Ficus altissima*** Blume | Moraceae | shrub | not evaluated | Guangxi |  | 1 | entrance zone |
| ***Ficus cyrtophylla*** (Wall. ex Miq.) Miq. | Moraceae | shrub | not evaluated | Guangxi |  | 1 | entrance zone |
| ***Ficus erecta*** Thunb. | Moraceae | shrub | not evaluated | Guizhou |  | 2 | entrance zone |
| ***Ficus glaberrima*** Blume | Moraceae | tree | not evaluated | Guangxi |  | 1 | entrance zone |
| ***Ficus hederacea*** Roxb. | Moraceae | shrub | not evaluated | Guangxi |  | 2 | entrance zone |
| ***Ficus pumila*** L. | Moraceae | shrub | not evaluated | Guangxi |  | 2 | entrance zone |
| ***Ficus sarmentosa*** Buch.-Ham. ex Sm. | Moraceae | shrub | not evaluated | Guangxi |  | 1 | entrance zone |
| ***Ficus superba*** Miq. | Moraceae | shrub | not evaluated | Guangxi |  | 2 | entrance zone |
| ***Ficus tinctoria*** G.Forst. | Moraceae | shrub | not evaluated | Guangxi |  | 3 | entrance zone |
| ***Fissistigma retusum*** (H.Lév.) Rehder | Annonaceae | Liana | not evaluated | Guangxi |  | 1 | entrance zone |
| ***Girardinia diversifolia*** (Link) Friis | Urticaceae | herb | not evaluated | Guizhou |  | 1 | entrance zone |
| ***Glabrella leiophylla*** (Fang Wen & Y.G.Wei) Fang Wen, Y.G. Wei & Mich. Möller | Gesneriaceae | herb | not evaluated | Guizhou |  | 1 | entrance zone |
| ***Glabrella mihieri*** (Franch.) Mich.Möller & W.H.Chen | Gesneriaceae | herb | not evaluated | Yunnan |  | 1 | entrance zone |
| ***Goodyera schlechtendaliana*** Rchb.f. | Orchidaceae | herb | LC | Yunnan |  | 1 | entrance zone |
| ***Gynostemma pentaphyllum*** (Thunb.) Makino | Cucurbitaceae | herb | not evaluated | Guangxi |  | 1 | entrance zone |
| ***Hedera sinensis*** (Tobler) Hand.-Mazz. | Araliaceae | herb | not evaluated | Guangxi, Guizhou |  | 2 | entrance zone |
| ***Helwingia japonica*** (Thunb.) F.Dietr. | Helwingiaceae | herb | not evaluated | Guizhou |  | 1 | entrance zone |
| ***Hemiboea gracilis*** Franch. | Gesneriaceae | herb | not evaluated | Guizhou |  | 1 | entrance zone |
| ***Hemiboea ovalifolia*** (W.T. Wang) A. Weber & M. Möller | Gesneriaceae | herb | not evaluated | Guizhou |  | 1 | entrance zone |
| ***Hemiboea sp1*** | Gesneriaceae | herb | not evaluated | Guangxi |  | 1 | entrance zone |
| ***Hemiboea subcapitata*** C.B. Clarke | Gesneriaceae | herb | not evaluated | Yunnan |  | 2 | entrance zone |
| ***Heteroplexis incana*** J.Y.Liang | Asteraceae | herb | not evaluated | Guangxi |  | 1 | entrance zone |
| ***Heterostemma alatum*** Wight & Arn. | Asclepiadaceae | Liana | not evaluated | Guangxi |  | 1 | entrance zone |
| ***Hoya sp1*** | Asclepiadaceae | herb | not evaluated | Guangxi |  | 1 | entrance zone |
| ***Impatiens apalophylla*** Hook. f. | Balsaminaceae | herb | not evaluated | Guangxi, Guizhou |  | 3 | entrance zone |
| ***Impatiens hunanensis*** Y.L. Chen | Balsaminaceae | herb | not evaluated | Guangxi |  | 2 | entrance zone |
| ***Impatiens kamtilongensis*** Toppin | Balsaminaceae | herb | not evaluated | Yunnan |  | 1 | entrance zone |
| ***Impatiens liboensis*** K. M. Liu & R. P. Kuang | Balsaminaceae | herb | not evaluated | Guizhou | only known from caves | 2 | entrance zone |
| ***Impatiens morsei*** Hook. f. | Balsaminaceae | herb | VU | Guangxi |  | 2 | entrance zone |
| ***Impatiens parvisepala*** S.X.Yu & Y.T.Hou | Balsaminaceae | herb | VU | Guangxi |  | 1 | entrance zone |
| ***Impatiens tianlinensis*** S. X. Yu & L. J. Zhang | Balsaminaceae | herb | not evaluated | Guizhou |  | 2 | entrance zone |
| ***Impatiens wenshanensis*** S.H. Huang | Balsaminaceae | herb | not evaluated | Yunnan |  | 1 | entrance zone |
| ***Iodes seguinii*** (H.Lév.) Rehder | Icacinaceae | herb | not evaluated | Guangxi |  | 1 | entrance zone |
| ***Isodon ternifolius*** (D.Don) Kudô | Lamiaceae | herb | not evaluated | Guizhou |  | 1 | entrance zone |
| ***Jasminum sp1*** | Oleaceae | herb | not evaluated | Guangxi |  | 1 | entrance zone |
| ***Justicia quadrifaria*** (Nees) T. Anderson | Acanthaceae | Liana | not evaluated | Guangxi |  | 1 | entrance zone |
| ***Laportea bulbifera*** (Siebold & Zucc.) Wedd. | Urticaceae | shrub | not evaluated | Guangxi |  | 1 | entrance zone |
| ***Laportea violacea*** Gagnep. | Urticaceae | herb | not evaluated | Guangxi |  | 1 | entrance zone |
| ***Lecanthus peduncularis*** (Wall. ex Royle) Wedd. | Urticaceae | herb | not evaluated | Yunnan |  | 1 | entrance zone |
| ***Lepisorus thunbergianus*** (Kaulf.) Ching | Polypodiaceae | fern | not evaluated | Guangxi |  | 1 | entrance zone |
| ***Lespedeza sp1*** | Fabaceae | herb | not evaluated | Guangxi |  | 1 | entrance zone |
| ***Ligustrum sinense*** Lour. | Oleaceae | herb | not evaluated | Guangxi |  | 1 | entrance zone |
| ***Lindenbergia philippensis*** (Cham. & Schltdl.) Benth. | Orobanchaceae | herb | not evaluated | Guangxi |  | 2 | entrance zone |
| ***Lindera sp1*** | Lauraceae | shrub | not evaluated | Guizhou |  | 1 | entrance zone |
| ***Liparis sp1*** | Orchidaceae | herb | not evaluated | Yunnan |  | 1 | entrance zone |
| ***Litsea elongata*** (Nees) Hook. f. | Lauraceae | shrub | not evaluated | Guizhou |  | 1 | entrance zone |
| ***Lodes seguinii*** (H.Lév.) Rehder | Icacinaceae | herb | not evaluated | Guangxi |  | 1 | entrance zone |
| ***Lonicera sp1*** | Caprifoliaceae | herb | not evaluated | Guangxi |  | 1 | entrance zone |
| ***Loxostigma griffithii*** (Wight) C.B. Clarke | Gesneriaceae | herb | not evaluated | Yunnan |  | 1 | entrance zone |
| ***Luculia pinceana*** Hook. | Rubiaceae | herb | not evaluated | Guangxi |  | 2 | entrance zone |
| ***Lycianthes biflora*** (Lour.) Bitter | Solanaceae | herb | not evaluated | Guangxi |  | 1 | entrance zone |
| ***Lysimachia filipes*** C.Z. Gao & D. Fang | Primulaceae | herb | not evaluated | Guangxi, Guizhou | only known from caves | 5 | entrance zone |
| ***Lysimachia saxicola*** Chun & F. H. Chun in F. H. Chen & C. M. Hu | Primulaceae | herb | not evaluated | Guangxi |  | 1 | entrance zone |
| ***Lysionotus denticulosus*** W.T. Wang | Gesneriaceae | herb | not evaluated | Guangxi, Guizhou |  | 3 | entrance zone |
| ***Lysionotus fengshanensis*** Yan Liu & D.X.Nong | Gesneriaceae | herb | not evaluated | Guangxi | only known from caves | 6 | entrance zone |
| ***Machilus sp1*** Yan Liu & D.X.Nong | Lauraceae | shrub | not evaluated | Guangxi |  | 1 | entrance zone |
| ***Maesa japonica*** (Thunb.) Moritzi & Zoll. | Primulaceae | herb | not evaluated | Guangxi |  | 1 | entrance zone |
| ***Magnolia championii*** Benth. | Magnoliaceae | shrub | not evaluated | Guangxi |  | 1 | entrance zone |
| ***Mahonia breviracema*** Y.S. Wang & P.G. Xiao | Berberidaceae | herb | not evaluated | Guangxi |  | 1 | entrance zone |
| ***Mazus pulchellus*** Hemsl. | Scrophulariaceae | herb | not evaluated | Guangxi |  | 1 | entrance zone |
| ***Microchirita hamosa*** (R.Br.) Yin Z.Wang | Gesneriaceae | herb | not evaluated | Guangxi,  Yunnan |  | 2 | entrance zone |
| ***Microsorum fortunei*** (T. Moore) Ching | Polypodiaceae | fern | not evaluated | Guangxi |  | 2 | entrance zone |
| ***Miliusa balansae*** Finet & Gagnep. | Annonaceae | shrub | not evaluated | Guizhou |  | 1 | entrance zone |
| ***Miliusa chunii*** Finet & Gagnep. | Annonaceae | shrub | not evaluated | Guangxi, Guizhou |  | 3 | entrance zone |
| ***Millettia sp1*** | Fabaceae | herb | not evaluated | Guangxi |  | 1 | entrance zone |
| ***Mitreola pingtaoi*** D. Fang & D.H. Qin | Loganiaceae | herb | not evaluated | Guangxi | only known from caves | 5 | entrance & twilight zone |
| ***Mitreola spathulifolia*** D. Fang & L.S. Zhou | Loganiaceae | herb | not evaluated | Guangxi |  | 1 | entrance zone |
| ***Morus mongolica*** (Bureau) C.K. Schneid. | Moraceae | herb | not evaluated | Yunnan |  | 1 | entrance zone |
| ***Myrioneuron faberi*** Hemsl. ex F.B.Forbes & Hemsl. | Rubiaceae | herb, shrub | not evaluated | Guangxi, Guizhou |  | 6 | entrance zone |
| ***Myrsine kwangsiensis*** (E. Walker) Pipoly & C. Chen | Primulaceae | shrub | not evaluated | Guangxi |  | 1 | entrance zone |
| ***Nandina domestica*** Thunb. | Berberidaceae | herb | not evaluated | Guangxi |  | 1 | entrance zone |
| ***Nanocnide lobata*** Wedd. | Urticaceae | herb | not evaluated | Guangxi |  | 1 | entrance zone |
| ***Neolepisorus ovatus*** Ching | Polypodiaceae | fern | not evaluated | Guangxi,  Yunnan |  | 2 | entrance zone |
| ***Nephrolepis cordifolia*** (L.) C. Presl | Nephrolepidaceae | fern | not evaluated | Guangxi |  | 5 | entrance zone |
| ***Ophiopogon sp1 'anlongensis'*** | Asparagaceae | herb | not evaluated | Guizhou | only known from caves | 1 | entrance zone |
| ***Ophiopogon sp2*** | Asparagaceae | herb | not evaluated | Guangxi, Guizhou |  | 2 | entrance zone |
| ***Ophiorrhiza japonica*** Blume | Rubiaceae | herb | not evaluated | Guangxi,  Yunnan |  | 4 | entrance zone |
| ***Ophiorrhiza sp1*** | Rubiaceae | herb | not evaluated | Guangxi |  | 4 | entrance zone |
| ***Ophiorrhiza sp2*** | Rubiaceae | herb | not evaluated | Guangxi |  | 3 | entrance zone |
| ***Ophiorrhiza sp3*** | Rubiaceae | herb | not evaluated | Yunnan |  | 1 | entrance zone |
| ***Ophiorrhiza sp4*** | Rubiaceae | herb | not evaluated | Guizhou |  | 1 | entrance zone |
| ***Oreocnide frutescens*** (Thunb.) Miq. | Urticaceae | shrub | not evaluated | Guangxi |  | 5 | entrance zone |
| ***Oreocnide kwangsiensis*** Hand.-Mazz. | Urticaceae | shrub | VU | Guangxi |  | 1 | entrance zone |
| ***Oreocnide sp1*** | Urticaceae | shrub | not evaluated | Guangxi |  | 1 | entrance zone |
| ***Ornithoboea wildeana*** W. G. Craib | Gesneriaceae | herb | not evaluated | Guangxi,  Yunnan |  | 3 | entrance zone |
| ***Oxalis griffithii*** Edgew. & Hook. f. | Oxalidaceae | herb | not evaluated | Guizhou |  | 1 | entrance zone |
| ***Oxyspora paniculata*** (D. Don) DC. | Melastomataceae | shrub | not evaluated | Guizhou |  | 1 | entrance zone |
| ***Paraboea nutans*** D. Fang & D.H. Qin | Gesneriaceae | herb | not evaluated | Guangxi |  | 1 | entrance zone |
| ***Paraboea peltifolia*** D. Fang & L. Zeng | Gesneriaceae | herb | not evaluated | Guangxi |  | 1 | entrance zone |
| ***Paraboea velutina*** (W.T. Wang & C.Z. Gao) B.L. Burtt | Gesneriaceae | herb | CR | Guangxi | only known from caves | 2 | entrance zone |
| ***Paraphlomis sp1*** | Lamiaceae | herb | not evaluated | Guizhou, Yunnan |  | 3 | entrance zone |
| ***Parthenocissus laetevirens*** Rehder | Vitaceae | herb | not evaluated | Guangxi, Guizhou |  | 6 | entrance zone |
| ***Passiflora papilio*** H.L. Li | Passifloraceae | Liana | not evaluated | Guangxi, Guizhou |  | 3 | entrance zone |
| ***Peliosanthes macrostegia*** Hance | Asparagaceae | herb | not evaluated | Guangxi, Guizhou |  | 3 | entrance zone |
| ***Pellionia heteroloba*** Wedd. | Urticaceae | herb | not evaluated | Guangxi |  | 2 | entrance zone |
| ***Pellionia leiocarpa*** W.T. Wang | Urticaceae | herb | not evaluated | Yunnan |  | 1 | entrance zone |
| ***Pellionia longzhouensis*** W.T.Wang | Urticaceae | herb | not evaluated | Guangxi | only known from caves | 1 | entrance zone |
| ***Persicaria chinensis*** (L.) H. Gross | Polygonaceae | herb | not evaluated | Guangxi, Guizhou |  | 3 | entrance zone |
| ***Petrocodon coccineus*** C.Y.Wu | Gesneriaceae | herb | not evaluated | Guangxi |  | 1 | entrance zone |
| ***Petrocodon dealbatus*** Hance | Gesneriaceae | herb | not evaluated | Guizhou |  | 1 | entrance zone |
| ***Petrocodon guangxiensis*** (Yan Liu & W.B. Xu) W.B. Xu & K.F. Chung | Gesneriaceae | herb | not evaluated | Guangxi | only known from caves | 1 | entrance zone |
| ***Petrocodon integrifolius*** (D. Fang & L. Zeng) A. Weber & M. Möller | Gesneriaceae | herb | not evaluated | Guangxi |  | 1 | entrance zone |
| ***Petrocodon jingxiensis*** (Y. Liu, H.S. Gao & W.B. Xu) A. Weber & M. Möller | Gesneriaceae | herb | not evaluated | Guangxi |  | 1 | entrance zone |
| ***Petrocodon lui*** (Y. Liu & W.B. Xu) A. Weber & M. Möller | Gesneriaceae | herb | not evaluated | Guangxi |  | 1 | entrance zone |
| ***Petrocodon sp1*** | Gesneriaceae | herb | not evaluated | Guangxi |  | 1 | entrance zone |
| ***Petrocosmea martini*** (H. Lév.) H. Lév. | Gesneriaceae | herb | not evaluated | Guangxi, Guizhou, Yunnan |  | 6 | entrance zone |
| ***Phyllagathis longearistata*** C. Chen | Melastomataceae | herb | not evaluated | Guangxi, Guizhou |  | 3 | entrance & twilight zone |
| ***Phyllocyclus lucidissimus*** (H.Lév. & Vaniot) Thiv | Gentianaceae | herb | not evaluated | Guangxi |  | 2 | entrance zone |
| ***Phymatosorus cuspidatus*** (D. Don) Pic. Serm. | Polypodiaceae | fern | not evaluated | Guizhou |  | 1 | entrance zone |
| ***Phytolacca acinosa*** Roxb. | Phytolaccaceae | herb | not evaluated | Guangxi |  | 1 | entrance zone |
| ***Pilea boniana*** Gagnep. | Urticaceae | herb | not evaluated | Guizhou, Guizhou, Yunnan |  | 8 | entrance zone |
| ***Pilea cavaleriei*** H.Lév. | Urticaceae | herb | not evaluated | Guangxi, Guizhou |  | 3 | entrance zone |
| ***Pilea cavernicola*** A.K. Monro, C.J. Chen & Y.G. Wei | Urticaceae | herb | not evaluated | Guangxi | only known from caves | 4 | entrance & twilight zone |
| ***Pilea japonica*** (Maxim.) Hand.-Mazz. | Urticaceae | herb | not evaluated | Guizhou |  | 3 | entrance zone |
| ***Pilea longicaulis*** Hand.-Mazz. | Urticaceae | herb | not evaluated | Guangxi |  | 6 | entrance zone |
| ***Pilea melastomoides*** (Poir.) Wedd. | Urticaceae | herb | not evaluated | Guangxi |  | 2 | entrance zone |
| ***Pilea notata*** C.H. Wright | Urticaceae | herb | not evaluated | Guangxi |  | 1 | entrance zone |
| ***Pilea peltata*** Hance | Urticaceae | herb | not evaluated | Guangxi |  | 2 | entrance zone |
| ***Pilea peploides*** (Gaudich.) Hook. & Arn. | Urticaceae | herb | not evaluated | Guangxi |  | 1 | entrance zone |
| ***Pilea plataniflora*** C.H. Wright | Urticaceae | herb | not evaluated | Guizhou |  | 1 | entrance zone |
| ***Pilea pseudonotata*** C.J. Chen | Urticaceae | herb | not evaluated | Guangxi |  | 1 | entrance zone |
| ***Pilea sp1*** | Urticaceae | herb | not evaluated | Guizhou, Yunnan | only known from caves | 2 | entrance zone |
| ***Pilea verrucosa*** Killip | Urticaceae | herb | not evaluated | Guangxi |  | 1 | entrance zone |
| ***Pinellia cordata*** N.E.Br. | Araceae | herb | not evaluated | Guangxi |  | 1 | entrance zone |
| ***Piper hancei*** Maxim. | Piperaceae | herb | not evaluated | Guangxi |  | 1 | entrance zone |
| ***Piper sp1*** | Piperaceae | herb | not evaluated | Guangxi |  | 3 | entrance zone |
| ***Piper sp2*** | Piperaceae | herb | not evaluated | Guizhou |  | 2 | entrance zone |
| ***Piper sp3*** | Piperaceae | herb | not evaluated | Guizhou |  | 1 | entrance zone |
| ***Pistacia weinmanniifolia*** J. Poiss. ex Franch. | Anacardiaceae | shrub | not evaluated | Guizhou |  | 1 | entrance zone |
| ***Pittosporum glabratum*** Lindl. | Pittosporaceae | herb | not evaluated | Guangxi |  | 1 | entrance zone |
| ***Pleurosoriopsis makinoi*** (Maxim. ex Makino) Fomin | Pleurosoriopsidaceae | fern | not evaluated | Guizhou |  | 1 | entrance zone |
| ***Polypodiodes amoena*** (Wall. ex Mett.) Ching | Polypodiaceae | fern | not evaluated | Yunnan |  | 1 | entrance zone |
| ***Polystichum acutidens*** Christ | Dryopteridaceae | fern | not evaluated | Guizhou |  | 1 | entrance zone |
| ***Polystichum articulatipilosum*** H.G. Zhou & Hua Li | Dryopteridaceae | fern | not evaluated | Guangxi | only known from caves | 2 | entrance zone |
| ***Polystichum craspedosorum*** (Maxim.) Diels | Dryopteridaceae | fern | not evaluated | Guizhou |  | 2 | entrance zone |
| ***Polystichum cyclolobum*** C. Chr. | Dryopteridaceae | fern | not evaluated | Guizhou |  | 1 | entrance zone |
| ***Polystichum deltodon*** (Baker) Diels | Dryopteridaceae | fern | not evaluated | Guizhou, Yunnan |  | 2 | entrance zone |
| ***Polystichum dielsii*** Christ | Dryopteridaceae | fern | not evaluated | Guangxi, Guizhou, Yunnan |  | 5 | entrance zone |
| ***Polystichum excellens*** Ching | Dryopteridaceae | fern | not evaluated | Guizhou |  | 2 | entrance & twilight zone |
| ***Polystichum excelsius*** Ching & Z.Y. Liu | Dryopteridaceae | fern | not evaluated | Yunnan |  | 1 | entrance zone |
| ***Polystichum fengshanense*** Li Bing Zhang & H. He | Dryopteridaceae | fern | not evaluated | Guangxi | only known from caves | 1 | entrance & twilight zone |
| ***Polystichum hecatopterum*** Diels | Dryopteridaceae | fern | not evaluated | Guizhou |  | 1 | entrance zone |
| ***Polystichum lanceolatum*** Baker | Dryopteridaceae | fern | not evaluated | Guizhou |  | 1 | entrance zone |
| ***Polystichum makinoi*** (Tagawa) Tagawa | Dryopteridaceae | fern | not evaluated | Guizhou |  | 1 | entrance zone |
| ***Polystichum minutissimum*** L.B. Zhang & H. He | Dryopteridaceae | fern | not evaluated | Guizhou | only known from caves | 1 | entrance zone |
| ***Polystichum sp1*** | Dryopteridaceae | fern | not evaluated | Guangxi |  | 2 | entrance zone |
| ***Polystichum sp3*** | Dryopteridaceae | fern | not evaluated | Guizhou |  | 1 | entrance zone |
| ***Polystichum sp4*** | Dryopteridaceae | fern | not evaluated | Guizhou |  | 4 | entrance zone |
| ***Polystichum sp5*** | Dryopteridaceae | fern | not evaluated | Guizhou |  | 2 | entrance zone |
| ***Pothos chinensis*** (Raf.) Merr. | Araceae | herb | not evaluated | Guangxi, Guizhou |  | 3 | entrance zone |
| ***Pouzolzia sanguinea*** (Blume) Merr. | Urticaceae | herb | not evaluated | Guangxi |  | 1 | entrance zone |
| ***Pouzolzia sp1*** | Urticaceae | shrub | not evaluated | Yunnan |  | 1 | entrance zone |
| ***Premna crassa*** Hand.-Mazz. | Verbenaceae | herb | not evaluated | Guangxi |  | 1 | entrance zone |
| ***Primula sp1*** | Primulaceae | herb | not evaluated | Yunnan |  | 1 | entrance zone |
| ***Primula wangii*** F.H. Chen & C.M. Hu | Primulaceae | herb | not evaluated | Guangxi |  | 3 | entrance zone |
| ***Primulina bipinnatifida*** (W.T.Wang) Yin Z.Wang & J.M.Li | Gesneriaceae | herb | not evaluated | Guangxi |  | 1 | entrance zone |
| ***Primulina carnosifolia*** (C.Y.Wu ex H.W.Li) Yin Z.Wang | Gesneriaceae | herb | not evaluated | Yunnan |  | 1 | entrance zone |
| ***Primulina debaoensis*** (Pellegr.) Mich.Möller & A.Weber | Gesneriaceae | herb | not evaluated | Guangxi | only known from caves | 1 | entrance zone |
| ***Primulina fengshanensis*** (Pellegr.) Mich.Möller & A.Weber | Gesneriaceae | herb | not evaluated | Guangxi | only known from caves | 1 | entrance zone |
| ***Primulina hezhouensis*** (W.H. Wu & W.B. Xu) W.B. Xu & K.F. Chung | Gesneriaceae | herb | not evaluated | Guangxi | only known from caves | 1 | entrance zone |
| ***Primulina laxiflora*** (W.T.Wang) Yin Z.Wang | Gesneriaceae | herb | not evaluated | Guangxi |  | 2 | entrance zone |
| ***Primulina liboensis*** (W.T.Wang & D.Y.Chen) Mich.Möller & A.Weber | Gesneriaceae | herb | not evaluated | Guangxi |  | 2 | entrance zone |
| ***Primulina lingchuanensis*** (Yan Liu & Y.G.Wei) Mich.Möller & A.Weber | Gesneriaceae | herb | not evaluated | Guangxi |  | 1 | entrance zone |
| ***Primulina luochengensis*** (Yan Liu & W.B.Xu) Mich.Möller & A.Weber | Gesneriaceae | herb | not evaluated | Guangxi |  | 1 | entrance zone |
| ***Primulina multifida*** (Pellegr.) Mich.Möller & A.Weber | Gesneriaceae | herb | not evaluated | Guangxi | only known from caves | 1 | entrance zone |
| ***Primulina nandanensis*** (S.X.Huang, Y.G.Wei & W.H.Luo) Mich.Möller & A.Weber | Gesneriaceae | herb | not evaluated | Guangxi, Guizhou |  | 3 | entrance zone |
| ***Primulina renifolia*** (D.Fang & D.H.Qin) J.M.Li & Yin Z.Wang | Gesneriaceae | herb | CR | Guangxi | only known from caves | 1 | entrance & twilight zone |
| ***Primulina repanda*** (W.T.Wang) Yin Z.Wang | Gesneriaceae | herb | not evaluated | Guangxi |  | 2 | entrance zone |
| ***Primulina shouchengensis*** (Z.Yu Li) Z.Yu Li | Gesneriaceae | herb | not evaluated | Guangxi |  | 1 | entrance zone |
| ***Primulina sp1*** | Gesneriaceae | herb | not evaluated | Guangxi |  | 1 | entrance zone |
| ***Primulina sp2 'xingpingensis'*** | Gesneriaceae | herb | not evaluated | Guangxi | only known from caves | 2 | entrance zone |
| ***Primulina tribracteata*** (W.T.Wang) Mich.Möller & A.Weber | Gesneriaceae | herb | not evaluated | Guangxi |  | 2 | entrance zone |
| ***Primulina weii*** Mich.Möller & A.Weber | Gesneriaceae | herb | not evaluated | Guangxi |  | 1 | entrance zone |
| ***Psychotria asiatica*** L. | Rubiaceae | herb | not evaluated | Guangxi |  | 1 | entrance zone |
| ***Psychotria prainii*** H.Lév. | Rubiaceae | herb | not evaluated | Guangxi |  | 2 | entrance zone |
| ***Pteridrys lofouensis*** (Christ) C. Chr. & Ching | Aspidiaceae | fern | not evaluated | Guizhou |  | 1 | entrance zone |
| ***Pteris actiniopteroides*** Christ | Pteridaceae | fern | not evaluated | Guangxi |  | 1 | entrance zone |
| ***Pteris angustipinnula*** Ching & S.H. Wu | Pteridaceae | fern | not evaluated | Guangxi |  | 1 | entrance zone |
| ***Pteris decrescens*** Christ | Pteridaceae | fern | not evaluated | Guizhou |  | 1 | entrance zone |
| ***Pteris deltodon*** Baker | Pteridaceae | fern | not evaluated | Guangxi, Guizhou |  | 5 | entrance zone |
| ***Pteris esquirolii*** Christ | Pteridaceae | fern | not evaluated | Yunnan |  | 1 | entrance zone |
| ***Pteris guangdongensis*** Ching | Pteridaceae | fern | not evaluated | Guangxi |  | 1 | entrance zone |
| ***Pteris henryi*** Christ | Pteridaceae | fern | not evaluated | Guangxi, Guizhou |  | 3 | entrance zone |
| ***Pteris linearis*** Poir. | Pteridaceae | fern | not evaluated | Guangxi |  | 2 | entrance zone |
| ***Pteris multifida*** Poir. | Pteridaceae | fern | not evaluated | Guangxi |  | 2 | entrance zone |
| ***Pteris sp1*** | Pteridaceae | fern | not evaluated | Guangxi |  | 3 | entrance zone |
| ***Pteris sp2*** | Pteridaceae | fern | not evaluated | Guangxi |  | 1 | entrance zone |
| ***Pteris sp3*** | Pteridaceae | fern | not evaluated | Guangxi |  | 1 | entrance zone |
| ***Pteris vittata*** L. | Pteridaceae | fern | LC | Guangxi |  | 1 | entrance zone |
| ***Pueraria montana*** (Lour.) Merr. | Fabaceae | herb | not evaluated | Guangxi |  | 1 | entrance zone |
| ***Reynoutria multiflora*** (Thunb.) Moldenke | Polygonaceae | herb | not evaluated | Guangxi, Guizhou |  | 3 | entrance zone |
| ***Rhapis humilis*** Blume | Arecaceae | herb | not evaluated | Guangxi, Guizhou |  | 4 | entrance zone |
| ***Rhynchoglossum obliquum*** Blume | Gesneriaceae | herb | not evaluated | Guizhou, Yunnan |  | 3 | entrance zone |
| ***Rosa cymosa*** Tratt. | Rosaceae | shrub | not evaluated | Guangxi |  | 1 | entrance zone |
| ***Rubus corchorifolius*** L.f. | Rosaceae | shrub | not evaluated | Guangxi |  | 2 | entrance zone |
| ***Rubus niveus*** Thunb. | Rosaceae | shrub | not evaluated | Guangxi |  | 1 | entrance zone |
| ***Rubus sp1*** | Rosaceae | shrub | not evaluated | Guizhou |  | 1 | entrance zone |
| ***Rubus tsangii*** Merr. | Rosaceae | shrub | not evaluated | Guangxi |  | 1 | entrance zone |
| ***Sabia discolor*** Dunn | Sabiaceae | herb | not evaluated | Guizhou |  | 1 | entrance zone |
| ***Sabia limoniacea*** Wall. ex Hook. f. & Thomson | Sabiaceae | Liana, shrub | not evaluated | Guangxi, Guizhou |  | 4 | entrance zone |
| ***Sabia parviflora*** Wall. | Sabiaceae | Liana | not evaluated | Guizhou |  | 1 | entrance zone |
| ***Sambucus javanica*** Blume | Caprifoliaceae | shrub | not evaluated | Guangxi |  | 1 | entrance zone |
| ***Sarcococca ruscifolia*** Stapf | Buxaceae | shrub | not evaluated | Guangxi, Guizhou |  | 5 | entrance zone |
| ***Sarcosperma pedunculatum*** Hemsl. | Sapotaceae | shrub | not evaluated | Guangxi |  | 1 | entrance zone |
| ***Sauropus sp1*** | Euphorbiaceae | herb | not evaluated | Guangxi |  | 1 | entrance zone |
| ***Saxifraga stolonifera*** Curtis | Saxifragaceae | herb | not evaluated | Guizhou |  | 1 | entrance zone |
| ***Schefflera bodinieri*** (H.Lév.) Rehder | Araliaceae | shrub | not evaluated | Guangxi, Guizhou, Yunnan |  | 3 | entrance zone |
| ***Schizocapsa plantaginea*** (Hance) Drenth | Taccaceae | herb | not evaluated | Guangxi |  | 1 | entrance zone |
| ***Scutellaria indica*** L. | Lamiaceae | shrub | not evaluated | Guangxi, Guizhou |  | 5 | entrance zone |
| ***Selaginella delicatula*** (Desv. ex Poir.) Alston | Selaginellaceae | fern | not evaluated | Guangxi, Guizhou |  | 5 | entrance zone |
| ***Selaginella doederleinii*** Hieron. | Selaginellaceae | fern | not evaluated | Guizhou |  | 1 | entrance zone |
| ***Selaginella moellendorffii*** Hieron. | Selaginellaceae | fern | not evaluated | Guangxi, Guizhou |  | 4 | entrance zone |
| ***Selaginella ornata*** Spring | Selaginellaceae | fern | not evaluated | Guangxi |  | 4 | entrance zone |
| ***Selaginella sp1*** | Selaginellaceae | fern | not evaluated | Guangxi, Guizhou, Yunnan |  | 4 | entrance zone |
| ***Selaginella sp3*** | Selaginellaceae | fern | not evaluated | Guangxi, Guizhou |  | 3 | entrance zone |
| ***Selaginella sp4*** | Selaginellaceae | fern | not evaluated | Guizhou, Yunnan |  | 3 | entrance zone |
| ***Selaginella uncinata*** (Desv. ex Poir.) Spring | Selaginellaceae | fern | not evaluated | Guangxi, Guizhou |  | 5 | entrance & twilight zone |
| ***Setaria palmifolia*** (J.Koenig) Stapf | Poaceae | herb | not evaluated | Guangxi |  | 1 | entrance zone |
| ***Solanum lyratum*** Thunb. | Solanaceae | herb | not evaluated | Guangxi, Guizhou |  | 2 | entrance zone |
| ***Speranskia tuberculata*** (Bunge) Baill. | Euphorbiaceae | herb | not evaluated | Guizhou |  | 2 | entrance zone |
| ***Spiradiclis longzhouensis*** H.S.Lo | Rubiaceae | herb | not evaluated | Guangxi |  | 4 | entrance zone |
| ***Spiradiclis tomentosa*** D. Fang & D.H. Qin | Rubiaceae | herb | not evaluated | Guangxi | only known from caves | 3 | entrance & twilight zone |
| ***Spiradiclis villosa*** X.X.Chen & W.L.Sha | Rubiaceae | herb | not evaluated | Guangxi |  | 1 | entrance zone |
| ***Sterculia lanceolata*** Cav. | Sterculiaceae | herb | not evaluated | Guangxi |  | 1 | entrance zone |
| ***Streptolirion volubile*** Edgew. | Commelinaceae | herb | not evaluated | Guizhou |  | 1 | entrance zone |
| ***Strobilanthes bantonensis*** Lindau | Acanthaceae | herb | not evaluated | Guangxi |  | 1 | entrance zone |
| ***Strobilanthes botryantha*** D. Fang & H.S. Lo | Acanthaceae | herb | not evaluated | Guangxi |  | 1 | entrance zone |
| ***Strobilanthes dimorphotricha*** Hance | Acanthaceae | herb | not evaluated | Guangxi |  | 1 | entrance zone |
| ***Strobilanthes sp1*** | Acanthaceae | herb | not evaluated | Guangxi, Guizhou |  | 2 | entrance zone |
| ***Strobilanthes sp2*** | Acanthaceae | herb | not evaluated | Guizhou |  | 2 | entrance zone |
| ***Strobilanthes sp3*** | Acanthaceae | herb | not evaluated | Guangxi |  | 1 | entrance zone |
| ***Tectaria devexa*** (Kunze) Copel. | Aspidiaceae | fern | not evaluated | Guangxi, Guizhou |  | 3 | entrance zone |
| ***Tectaria gemmifera*** (Fée) Alston | Aspidiaceae | fern | not evaluated | Yunnan |  | 1 | entrance zone |
| ***Tectaria sp1*** | Aspidiaceae | fern | not evaluated | Guizhou |  | 1 | entrance zone |
| ***Tectaria subpedata*** (Harr.) Ching | Aspidiaceae | herb | not evaluated | Guangxi |  | 1 | entrance zone |
| ***Tetrapanax papyrifer*** (Hook.) K.Koch | Araliaceae | shrub | not evaluated | Guangxi |  | 2 | entrance zone |
| ***Thalictrum ichangense*** Lecoy. ex Oliv. | Ranunculaceae | herb | not evaluated | Guangxi |  | 2 | entrance zone |
| ***Tirpitzia ovoidea*** Chun & F.C. How ex W.L. Sha | Linaceae | herb | not evaluated | Guangxi |  | 1 | entrance zone |
| ***Trachelospermum jasminoides*** (Lindl.) Lem. | Apocynaceae | herb | not evaluated | Guangxi, Guizhou |  | 5 | entrance zone |
| ***Trichosanthes sp1*** | Cucurbitaceae | herb | not evaluated | Guangxi |  | 1 | entrance zone |
| ***Urtica fissa*** E. Pritz. | Urticaceae | herb | not evaluated | Guangxi |  | 1 | entrance zone |
| ***Urtica mairei*** H.Lév. | Urticaceae | herb | not evaluated | Guizhou |  | 1 | entrance zone |
| ***Vernonia sp1*** | Asteraceae | herb | not evaluated | Guangxi |  | 1 | entrance zone |
| ***Viola fargesii*** H. Boissieu | Violaceae | herb | not evaluated | Guangxi, Guizhou |  | 2 | entrance zone |
| ***Viola inconspicua*** Blume | Violaceae | herb | not evaluated | Guangxi |  | 1 | entrance zone |
| ***Viola sp1*** | Violaceae | herb | not evaluated | Guizhou |  | 1 | entrance zone |
| ***Zanthoxylum armatum*** DC. | Rutaceae | tree | not evaluated | Guangxi |  | 1 | entrance zone |
| ***Zanthoxylum sp2*** | Rutaceae | tree | not evaluated | Guangxi |  | 1 | entrance zone |
