# Supplementary material for: Discovery of a diverse cave flora in China
Source: PLoS One. 2018 Feb 7;13(2):e0190801. doi: 10.1371/journal.pone.0190801 (PMC5802439; doi:10.1371/journal.pone.0190801)
Supplement: S3 Table — Comparison based on the use of a complete assessment of the Gesneriaceae angiosperm family. Source, Gesneriaceae of South China, Nanning: Guangxi Science and Technology Publishing House, Nanning; 2010. (DOCX) [file pone.0190801.s003.docx]

**S3 Table. A comparison of the frequency of threatened species in cave and non-cave habitats.** Comparison based on the use of a complete assessment of the Gesneriaceae angiosperm family. Source, Gesneriaceae of South China, Nanning: Guangxi Science and Technology Publishing House, Nanning; 2010.

|  | CR | EN | VU | Not Threatened | Total Threatened (CR-VU) |
| --- | --- | --- | --- | --- | --- |
| Species observed in caves (n) | 40% (17) | 7% (3) | 9% (4) | 44% (19) | 56% (24) |
| Species documented in S China’ (n) | 40% (122) | 8% (25) | 6% (19) | 46% (139) | 57% (166) |
| Cave-restricted species (n) | 70% (7) | 0% (0) | 0% (0) | 30% (3) | 70% (7) |
