# Supplementary figures and images for: Discovery of a diverse cave flora in China
Source: PLoS One. 2018 Feb 7;13(2):e0190801. doi: 10.1371/journal.pone.0190801 (PMC5802439; doi:10.1371/journal.pone.0190801)

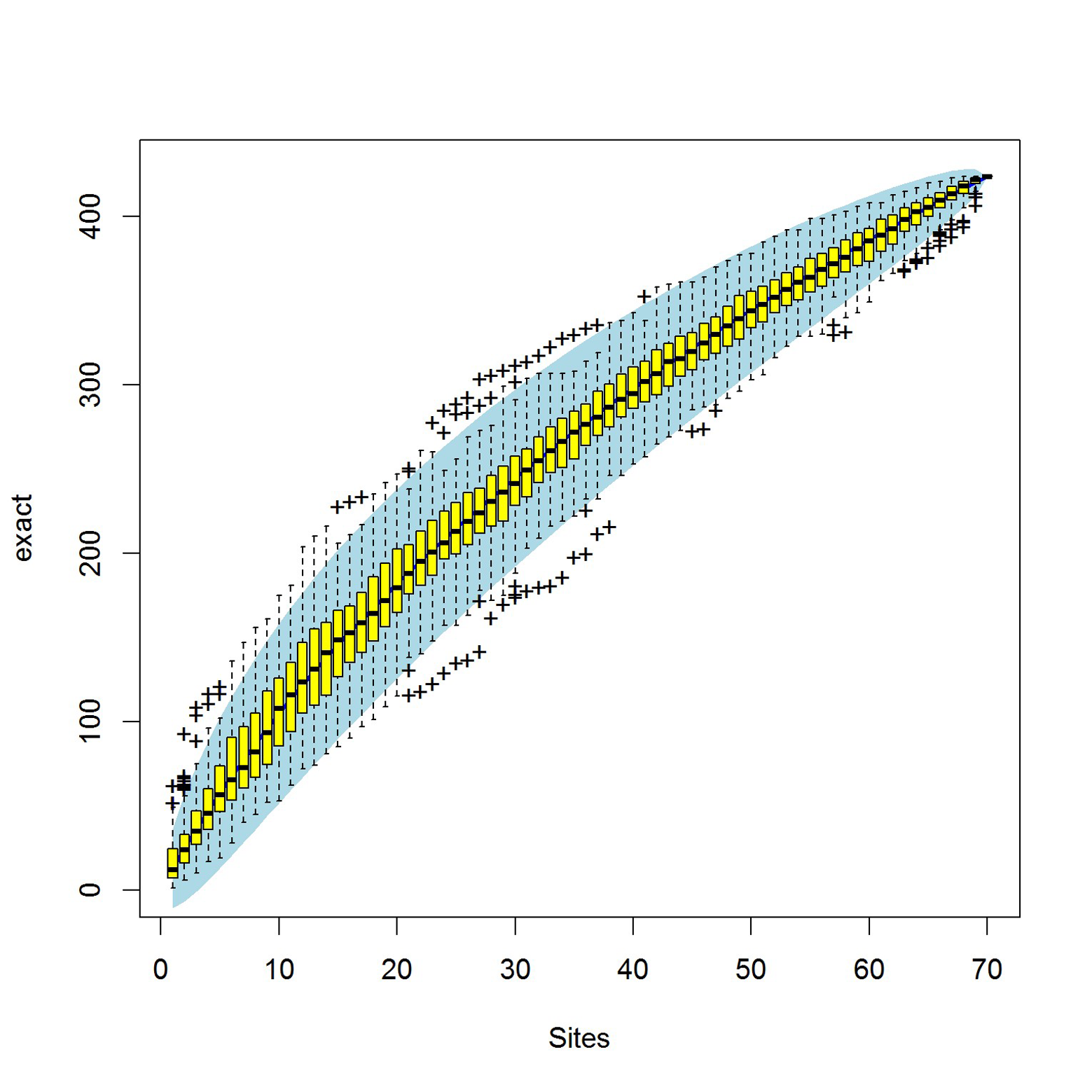

Supplement: S1 Fig — The SAC produced by “exact” method is shown in blue; the SAC produced by “random” method is shown as yellow boxes with outlies as black crosses. (TIF) [file pone.0190801.s004.tif]
